# *Culicoides* Midge Abundance across Years: Modeling Inter-Annual Variation for an Avian Feeder and a Candidate Vector of Hemorrhagic Diseases in Farmed Wildlife

**DOI:** 10.3390/v16050766

**Published:** 2024-05-11

**Authors:** Jamie S. Benn, Jeremy P. Orange, Juan Pablo Gomez, Emily T. N. Dinh, Bethany L. McGregor, Erik M. Blosser, Nathan D. Burkett-Cadena, Samantha M. Wisely, Jason K. Blackburn

**Affiliations:** 1 Spatial Epidemiology & Ecology Research Laboratory, Department of Geography, University of Florida, 3141 Turlington Hall, Gainesville, FL 32611, USA; jbenn@uwalumni.com (J.S.B.); jporange2@ufl.edu (J.P.O.); 2 Emerging Pathogens Institute, University of Florida, 2055 Mowry Road, Gainesville, FL 32611, USA; 3 Departamento de Química y Biología, Universidad del Norte, Barranquilla 080001, Colombia; echeverrip@uninorte.edu.co; 4 Michigan Department of Health and Human Services, 333 S Grand Ave, Lansing, MI 48933, USA; dinhe@michigan.gov; 5 USDA-ARS-Center for Grain and Animal Health Research-Arthropod-Borne Animal Diseases Research Unit, 1515 College Ave, Manhatten, KS 66506, USA; bethany.mcgregor@usda.gov; 6 Sutter-Yuba Mosquito & Vector Control District, 701 Bogue Road, Yuba City, CA 95991, USA; erik.blosser@gmail.com; 7 Florida Medical Entomology Laboratory, University of Florida, 200 9th St SE, Vero Beach, FL 32962, USA; nburkettcadena@ufl.edu; 8 Department of Wildlife Ecology and Conservation, 110 Newins-Ziegler Hall, Gainesville, FL 32611, USA; wisely@ufl.edu

**Keywords:** epizootic hemorrhagic disease virus, bluetongue virus, hemorrhagic disease, occupancy model, *Culicoides haematopotus*, *Culicoides stellifer*, *Culicoides venustus*, spatial model, white-tailed deer, disease transmission

## Abstract

(1) Background: Epizootic hemorrhagic disease virus (EHDV) and bluetongue virus (BTV) are orbiviruses that cause hemorrhagic disease (HD) with significant economic and population health impacts on domestic livestock and wildlife. In the United States, white-tailed deer (*Odocoileus virginianus*) are particularly susceptible to these viruses and are a frequent blood meal host for various species of *Culicoides* biting midges (Diptera: Ceratopogonidae) that transmit orbiviruses. The species of *Culicoides* that transmit EHDV and BTV vary between regions, and larval habitats can differ widely between vector species. Understanding how midges are distributed across landscapes can inform HD virus transmission risk on a local scale, allowing for improved animal management plans to avoid suspected high-risk areas or target these areas for insecticide control. (2) Methods: We used occupancy modeling to estimate the abundance of gravid (egg-laden) and parous (most likely to transmit the virus) females of two putative vector species, *C. stellifer* and *C. venustus*, and one species, *C. haematopotus*, that was not considered a putative vector. We developed a universal model to determine habitat preferences, then mapped a predicted weekly midge abundance during the HD transmission seasons in 2015 (July–October) and 2016 (May–October) in Florida. (3) Results: We found differences in habitat preferences and spatial distribution between the parous and gravid states for *C. haematopotus* and *C. stellifer*. Gravid midges preferred areas close to water on the border of well and poorly drained soil. They also preferred mixed bottomland hardwood habitats, whereas parous midges appeared less selective of habitat. (4) Conclusions: If *C. stellifer* is confirmed as an EHDV vector in this region, the distinct spatial and abundance patterns between species and physiological states suggest that the HD risk is non-random across the study area.

## 1. Introduction

Epizootic hemorrhagic disease virus (EHDV) and bluetongue virus (BTV) are globally distributed orbiviruses presenting important threats to livestock and wildlife health. Both viruses are known to infect cattle and sheep with outbreaks occurring in both North America and Europe [1,2]. Typically, these viruses circulate in stable enzootic/epizootic patterns [3] with subclinical infections or mild-to-moderate febrile disease in domestic herds [1,4], but in North America, EHDV and BTV have been found to consistently cause disease in wild ruminants where outbreaks in white-tailed deer (*Odocoileus virginianus*) [5,6,7], pronghorn (*Antilocapra americana*) [8,9], and bighorn sheep (*Ovis canadensis*) [6,10] have resulted in high morbidity and mortality.

White-tailed deer (WTD), the most abundant wild ruminant in North America, are particularly susceptible to multiple serotypes of EHDV and BTV [3,11,12,13,14]. These viruses present similarly in WTD and together are referred to as hemorrhagic disease (HD) [3]. Clinical signs of HD are characterized by high fever, respiratory distress, severe head and neck edema, hemorrhaging in body tissues, and cracked hooves. Acute infections can result in death within 8–36 h without clinical signs [4]. It is currently estimated that WTD in risk areas have a 29% infection with a 20% mortality rate [4,15]. WTD are also the most common species of farmed native and exotic wildlife [16,17]. WTD farming is an important industry in rural areas in the United States like Texas where antibodies against HD were detected in greater than 80% of surveyed farmed deer [18]. Given this high level of seroprevalence, WTD susceptibility to HD presents an ever-increasing economic and population health risk across North America and warrants research supporting disease management and prevention methods [1,3,4,7].

EHDV and BTV are vector-borne viruses transmitted by *Culicoides* (Diptera: Ceratopogonidae) midges with a few confirmed and several suspected competent *Culicoides* spp. vectors in different regions; virus distributions are linked to the geographic range of vectors (though disease distributions reflect multiple virus serotypes and vectors) [3]. In Florida, where HD causes significant mortality in WTD, several *Culicoides* spp. are suspected of transmitting EHDV and BTV. The larval ecology of *Culicoides* species varies widely, from wet treeholes to seepages and stream margins [19,20], making it challenging to clearly delineate the fine scale and local distribution of these viruses based on the distribution of putative vectors.

The range of these viruses is expanding, potentially through a mix of additional competent *Culicoides* species transported from other regions, expanded *Culicoides* vector range, and/or altered vector ecology due to climate change [21,22]. In North America, recent HD outbreaks have been reported in new regions of Canada that did not historically have HD [23,24]. At the same time, exotic serotypes of EHDV and BTV have been introduced to native vector species. For example, a previously unknown serotype of EHDV was recently discovered in North America [12,25], and a sub-Saharan serotype of BTV was introduced into an indigenous vector species in northern Europe [26,27,28,29]. How exotic serotypes are introduced to new regions remains unclear, although wind currents are one of the suspected mechanisms involved in midge dispersal [30]. Combining these unknowns with the expanding distributions of these viruses puts both naïve domestic livestock and wildlife species in previously unaffected areas at risk of more severe and more frequent outbreaks. Current research efforts by ours and various other teams around the world [31,32,33] are working to define the ecology of *Culicoides* vectors, namely their seasonal abundance, host, and habitat preferences, as identifying these details can help understand the expansion of HD viruses [34]. With this knowledge, we may be able to proactively manipulate herd habitat use to avoid specific areas at times of high vector abundance [35,36].

Here, we modeled spatially explicit *Culicoides* abundance on a local landscape over two years in the Florida panhandle. We evaluated how the site-specific abundance of several *Culicoides* species at the parous and gravid physiological states are related to different habitat types over repeated sampling events during the transmission seasons in 2015 and 2016 on a well-studied wildlife ranch. Our objectives were (1) to develop a universal model to predict the abundance of *Culicoides* spp. across years and states, (2) assess whether the physiological states of different species of *Culicoides* have contrasting habitat preferences, and (3) determine if abundance and habitat preferences are similar across multiple years. Lastly, we used the resulting model to predict and map the abundance of three *Culicoides* spp. during the HD transmission seasons to test our hypothesis that different species in different states prefer different habitats.

## 2. Materials and Methods

### 2.1. Study Area

This study was conducted on a 180 ha privately owned, high-fenced ranch in Gadsden County, Florida. The property was divided into a 172 ha hunting preserve and an 8 ha deer breeding facility. In the hunting preserve, there were between 130–150 free-ranging WTD and approximately 150 exotic cervids and bovids of 13 different species, resulting in an animal density of approximately 1.48 animals/ha that was managed with 12 supplementary protein feeders that were regularly filled by ranch personnel [37,38]. The animals had access to seven food plots across the ranch, although the timing of planting and food availability varied between years, and palatable vegetation was often low during the summer months during the study period. Water features on the ranch included one large pond (2.3 ha), two small ponds (0.35 ha and 0.1 ha), and a permanent stream fed by spring seepages. There were also 10 double-fenced WTD breeding pens within a larger exclusion zone totaling ~9.3 ha [34]; hunting preserve animals were excluded from the entire breeding pen area. The primary habitat type of the ranch was hardwood hammock with upland short-leaf pine species, such as loblolly pine (*Pinus taeda*), growing throughout the ranch [34].

### 2.2. Entomological Sampling

Complete entomological trapping methods are detailed in McGregor et al. [38] and Dinh et al. [34] Briefly, 20 spatially random trap locations were selected to represent all habitat types present on the ranch. Each site was equipped with a miniature CDC light trap (Model 2836BQ, BioQuip, Rancho Dominguez, CA, USA) and a blacklight-emitting diode array (Model 2790 V390, BioQuip, Rancho Dominguez, CA, USA), and controlled by a timer to operate starting 1 h prior to sunset to 1 h after sunrise. Samples were collected twice weekly between July 2015–October 2015 and May 2016–October 2016. Trapped midges were identified to species according to Blanton and Wirth [39] and categorized as nulliparous (never bloodfed), bloodfed (engorged with host blood), gravid (laden with eggs), or parous (having previously laid at least one batch of eggs). Resulting counts for *Culicoides* species at each physiological state were grouped by sampling week, and then count data were filtered to only those samples collected during the suspected HD season (May–October). This limited the data for analysis in this study to samples collected from July to October 2015 and May to October 2016. Histograms were created to visualize filtered count data and identify the most abundant species collected during the 2015 and 2016 seasons for comparative analysis across years and species with large enough sample sizes. Data were then filtered to counts of parous (females seeking blood meals after completing a gonotrophic cycle) and gravid (females carrying eggs) midges, as these states are most likely to transmit viruses through subsequent blood meals [40].

### 2.3. Environmental Data

**Figure 1:**
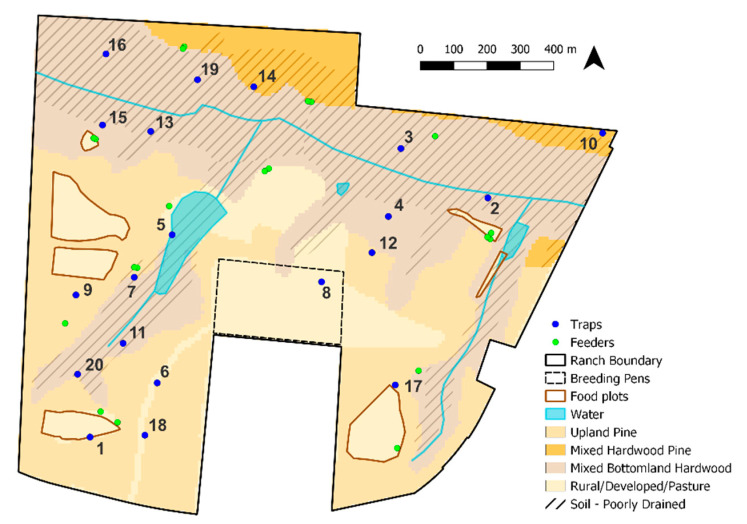
Map of study ranch in Gadsden County, Florida, illustrating the numbered trap locations, feeder locations, and all environmental variables considered in the current study. Poorly drained soil is identified by the lined area with the rest of the ranch categorized as well-drained soil. Numerals indicate trap site number.

Seven environmental covariates were derived, rasterized, and resampled to a 10 m cell size and clipped to the extent of the ranch (Table 1, Figure 1). We used the week in the HD season to account for time when developing our models and accounted for space by including the standardized latitude-longitude coordinates of the center of each 10 m raster cell containing a trap site. Additional covariates were included to investigate if different *Culicoides* species preferred different environmental factors at different physiological states. Similar to previous work detailed in Dinh et al. [34], the Euclidian distances from the nearest feeder and the nearest water body were included as proxies for the availability of potential blood meal hosts, such as WTD, and potential oviposition sites, respectively. Habitat type was included as unordered factor levels based on land cover data reported on the Cooperative Land Cover map (version 3.2) from the Florida Fish and Wildlife Conservation Commission (FWC) and the Florida Natural Areas Inventory (FNAI) [41] and was reclassified as defined in Dinh et al. [34,42] as upland pine covering 35.29% of the ranch, mixed hardwood pine covering 6.91% of the ranch, mixed bottomland hardwood covering 43.50% of the ranch, or rural/developed/pasture covering 14.29% of the ranch. A soil survey of the ranch was downloaded from the US Department of Agriculture’s Natural Resources Conservation Service (NRCS) Web Soil Survey application [43] and grouped by the Natural Drainage Class designation because *Culicoides* spp. larvae typically occur in moist and muddy substrates [39,44,45]. Soil types with Map Unit Symbols (MUSYMs) 6, 9, 31, and 36 were classified as well-drained, and soil types with MUSYMs 66, 86, and 88 were classified as poorly drained. Lastly, we included weekly utilization distributions (UD) [46,47] from 15 WTD, 1 fallow deer (*Dama dama*), and 1 Père David’s deer (*Elaphurus davidianus*) that were collared in a previous study on the same ranch during the midge sampling effort [42,48] to represent the probability of animal presence in the study environment regardless of the environmental characteristics or proximity to feeders [34]. These species were confirmed as preferred bloodmeals for midges on this ranch [38]. For this analysis, collared animals were resampled from 15 or 30 min intervals to 6 hours using the T-LoCoH R package. The kernelUD function from the adehabitatHR R package was then used to estimate a weekly UD for all movements combined. Kernel density estimates require the computation of a bandwidth. Here, we used least-squares cross-validation (LSCV) and calculated an average LSCV for all weeks as the bandwidth to create UDs per week. Kernel density estimates were output to the same 10 m raster surface as the environmental covariates.

We assessed the potential correlation between variables to avoid multicollinearity and model overfit by computing Pearson correlation coefficients (*r*) between numerical variables and ANOVAs between pairs of numerical and categorical variables in R. We did not find any correlation between the numerical variables as none of the Pearson’s r values were greater than |0.7| (Table S1), and there was no correlation between the numerical and categorical variables except for UD and soil (Table S2).

### 2.4. Model Construction

Using the workflow developed by Dinh et al. [34], environmental covariate values were extracted for each of the 20 trap sites. Continuous variables were standardized, and repeated count models were developed using the ‘unmarked’ R package version 1.1.1 [49,50]. Repeated count models were fitted with covariates as site-level covariates and a negative binomial prior mixing distribution because insects were not randomly distributed in space, and this distribution allows the density of animals to vary spatially [51]. Models were constructed based on the N-mixture model presented by Royle [52], which reasons that individual midges are always available to collect and assumes that a lack of collection suggests non-detectability or an apparent absence. This was applicable to our study because midge abundance, and therefore the likelihood of detection, is likely unaffected by repeated sampling events. Within our models, we used static site-level covariates to define abundance because other time-specific survey-level covariates (such as local weather conditions) were unavailable at the study ranch for the fine spatial resolution used here (10 m^2^ raster resolution).

First, we determined if counts were temporally and/or spatially dependent through null models that tested all variations of sampling week and trap coordinates as covariates. Null models also served to identify which distribution was most appropriate for the data: Poisson, negative binomial, or zero-inflated Poisson random variable. The most representative null model using the negative binomial distribution then served as the base for the development of 31 alternative models with different combinations of variables (week, latitude, longitude, feeder, water, habitat, soil, UD (Table S1)) for either the parous or gravid state. All 31 models were run for each species by physiological state for each year and were ranked separately by AIC. (Table 2). From the resulting list of best models for each species/state/year combination, the most common best model (GlobalE) was identified as the ‘universal model’ and used to predict the weekly abundance of biting midges of all three species for each life stage across the study ranch throughout the 2015 and 2016 transmission seasons, with additional predictions using the individual species/state/year best models provided in the supplement. Maps of predicted abundance were created in R and exported as JPEGs to create weekly GIF animations. Lastly, we visualized each model’s goodness of fit by plotting the actual counts of each species at each state against the predicted counts at each trap location over the duration of the sampling periods.

## 3. Results

### 3.1. Entomological Sampling

**Figure 2:**
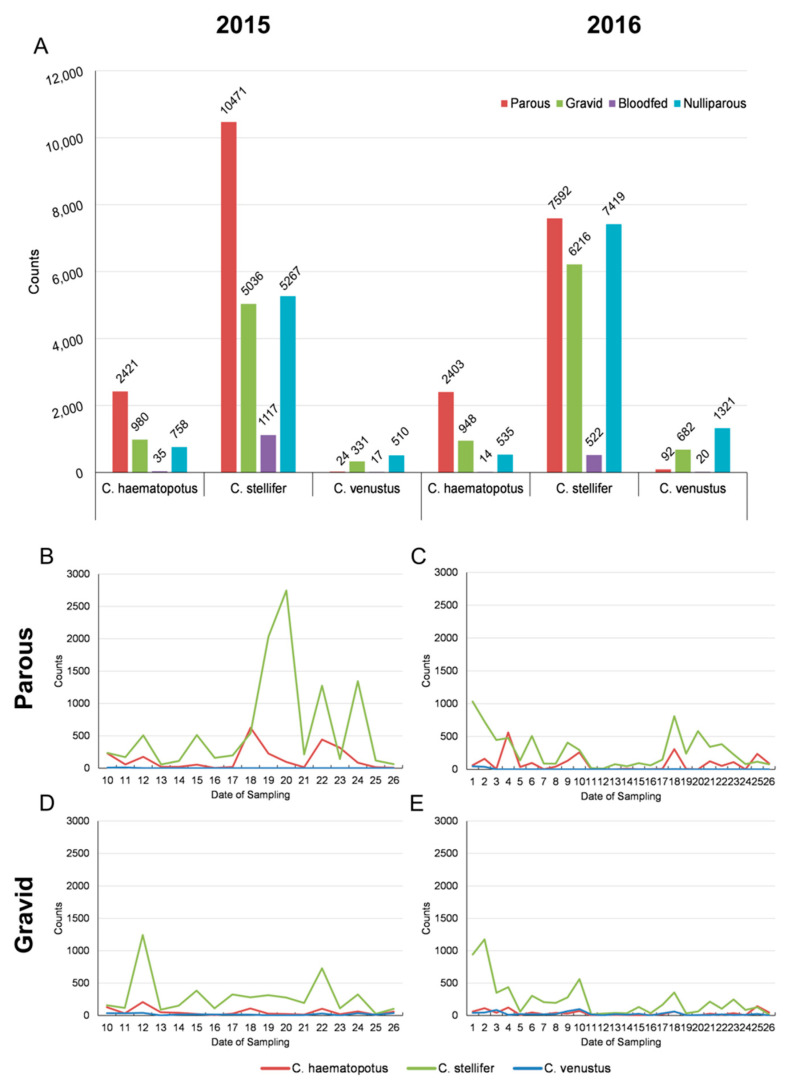
The total number of females of the three most abundant species (*Culicoides haematopotus*, *C. stellifer*, *C. venustus*) collected on a wildlife ranch in the Florida panhandle during 2015 (**left**) and 2016 (**right**) HD seasons at the parous, gravid, bloodfed, and nulliparous life stages (**A**) and the relative abundance of parous (**B**,**C**) and gravid (**D**,**E**) life stages throughout each season. Here, each week is reported from the first week of sampling in May forward to October. In 2015, sampling started in July (week 10), and in 2016, sampling started in May.

Entomological data collected between July–October 2015 and May–October 2016 are summarized in Figure S1 and Table S4. The most abundant species that were collected in both 2015 and 2016 are *C. stellifer*, *C. haematopotus*, and *C. venustus* (Figure S1). In 2015, a total of 28,887, and in 2016, a total of 29,296 midges were trapped and identified as either *C. haematopotus*, *C. stellifer* or *C. venustus* with counts subdivided by species and physiological status as parous, gravid, bloodfed, or nulliparous (Figure 2A). Here, only the parous and gravid categories are considered, with a relative abundance of each life stage for each species illustrated over time in Figure 2B.

### 3.2. Universal Model for Culicoides Species Abundance

Null models were run with different combinations of week and standardized latitude and longitude variables. For 2016, the best null model includes week and location, whereas the best null model for 2015 only includes location. Although time was not significant for the 2015 data, the null model that included week, latitude, and longitude was used for 31 alternative models to ensure we captured the variation of the HD season.

We then applied the 31 alternative models to evaluate three *Culicoides* species at the parous and gravid states for two years and encountered differences in the resulting best models for each of the 12 situations. With our priority being to draw comparisons among species, state, and year, we identified our universal model (GlobalE) as the most common, best model among the 12 best models identified, regardless of the ΔAIC > 2 for some situations (Table 2).

The universal model identified to predict *C. haematopotus*, *C. stellifer*, and *C. venustus* over two years at the parous and gravid states returned coefficient estimates for all covariates included in this study, as summarized in Table 3. Like predictions from running null models, all situations (*C. haematopotus* parous, *C. haematopotus* gravid, etc.) were significantly affected by week in 2016, with abundance decreasing throughout the season, while time had little effect on abundance in 2015, with only *C. haematopotus* gravid samples significantly decreasing over the season. When considering habitat, *C. haematopotus* gravid in 2016, *C. stellifer* gravid in 2015 and 2016, and *C. venustus* gravid in 2015 and 2016 have significant positive coefficients for mixed bottomland hardwood habitats. Across *C. haematopotus* and *C. stellifer* for both years, parous midge abundance appears to be marginally affected by mixed bottomland hardwood habitats, as only the *C. stellifer* parous samples in 2015 were significantly reduced in this habitat. In both years, across species or states, strong patterns were observed for distance to supplementary feeders and permanent water sources. Negative coefficients for both variables imply higher midge abundance with closer proximity to these features, particularly for gravid midges. Proximity to water may suggest higher midge abundance; however, there is also a positive correlation for well-drained soil for both parous and gravid *C. haematopotus* in 2015 and 2016, both parous and gravid *C. stellifer* in only 2016, and only gravid *C. venustus* in both 2015 and 2016. Lastly, only *C. haematopotus* parous in 2016 and, in 2015, *C. stellifer* and *C. venustus* gravid had significant correlations relating abundance to deer utilization of the area (UD).

Species-specific differences in covariates are more ambiguous, likely because of sample size differences among species. The most prominent species difference is with *C. haematopotus* for which both parous and gravid abundance for 2015 (β = 1.2017 and 0.4277, respectively) and 2016 (β = 1.0429 and 0.2375, respectively) is significantly increased with higher longitude, while *C. stellifer* parous abundance decreases with longitude for both years (β = −0.3199 and –0.4059, respectively). *Culicoides stellifer* and *C. venustus* gravid abundances increase in both 2015 (β = 1.3422 and 1.4783) and 2016 (β = 2.5083 and 2.3591) in mixed bottomland hardwood habitats. *Culicoides haematopotus*, as well as *C. stellifer* gravid abundance, are also significantly reduced in rural/developed/pasture habitats in both years. In general, all three species in both states have increased abundance closer to supplementary feeders, closer to permanent bodies of water and in habitats with well-drained soil, while abundances did not seem to be strongly affected by UD (Table 3).

The universal model was considered the best model (ΔAIC = 0) for 2015 *C. haematopotus* gravid, 2016 *C. haematopotus* parous and gravid, and 2016 *C. stellifer* gravid; however, it factored in all covariates (week, latitude, longitude, habitat, feeder, water, soil, and UD) when predicting *Culicoides* midge abundance. This resulted in higher ΔAICs for the universal model in situations with limited sample sizes, such as parous *C. venustus* samples in 2015, which had an ΔAIC of 8.3 (Table 2). For this reason, we developed secondary best models for the remaining combinations of year, species, and states for which the universal model was not the most parsimonious (Table S5). Furthermore, we opted to exclude all predictions for 2015 *C. venustus* parous from our analysis because the sample size was too small to generate representative models.

### 3.3. Spatial Predictions Using the Universal Model

**Figure 3:**
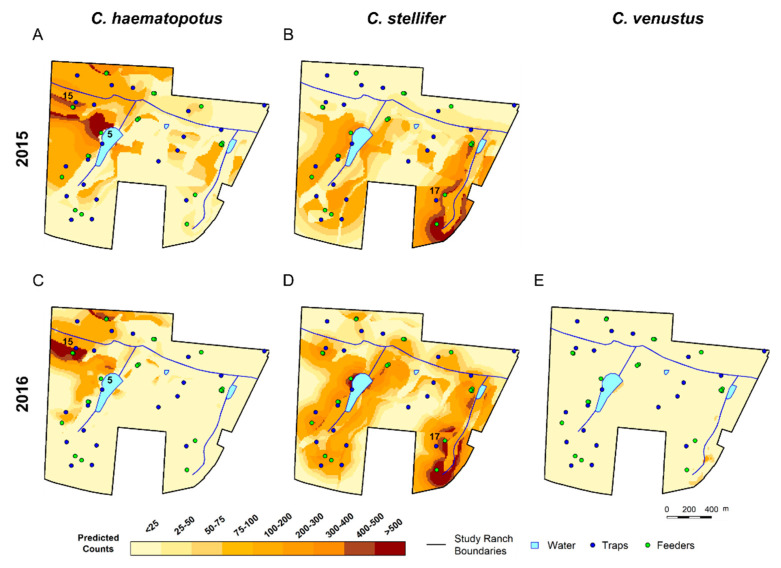
Predicted parous *Culicoides* abundance on a wildlife ranch in the Florida panhandle during the 14th week of the HD transmission season using the universal model to make predictions for *C. haematopotus* (**A**) and *C. stellifer* (**B**) in 2015 and predictions for *C. haematopotus* (**C**), *C. stellifer* (**D**), and *C. venustus* (**E**) in 2016. Also noted are the locations of CDC light traps 5 and 15 (**A**,**C**) and 17 (**B**,**D**). Counts for *C. venustus* were too low to use for spatial predictions in 2015.

**Figure 4:**
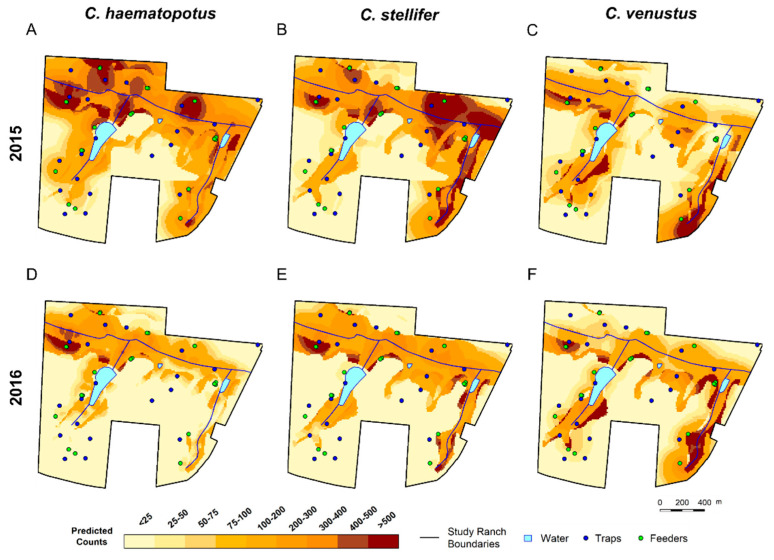
Predicted gravid *Culicoides* abundance on the study ranch during the 14th week of the HD transmission season using the universal model to make predictions for *C. haematopotus* (**A**), *C. stellifer* (**B**), and *C. venustus* (**C**) in 2015, and predictions for *C. haematopotus* (**D**), *C. stellifer* (**E**), and *C. venustus* (**F**) in 2016.

For simplicity, we have displayed the spatial predictions for the 14th week of the 2015 and 2016 hemorrhagic disease seasons using the universal model to predict *C. haematopotus*, *C. stellifer*, and *C. venustus* abundance at the parous (Figure 3) and gravid (Figure 4) states (GIFs for the entire season are animated in Figure S2 for 2015 and Figure S3 for 2016). The 14th week was approximately representative of the average midge counts for each species for the entire season. The clearest spatial difference observed in predictions with the universal model is that parous *C. haematopotus* would be most abundant on the northwestern section of the ranch close to traps 5 and 15 (Figure 3A), particularly close to the largest permanent body of water, while parous *C. stellifer* abundance is consistently highest on the southeastern side of the ranch near trap 17 distributed along the creek boundary (Figure 3B). Similar spatial patterns are observed in the predicted abundance for *C. haematopotus* and *C. stellifer* in their gravid states, but both species are predicted to have a wider geographic range in these general areas than in their parous states (Figure 4). These differences in spatial patterns are similar in both years, with the range for *C. haematopotus* concentrated in the northwest corner of the ranch, while *C. stellifer* occupied the length of both creeks running approximately north to south.

Parous midges of both *C. haematopotus* and *C. stellifer* were expected to populate areas with primarily upland pine habitats, with the greatest abundance predicted along the border of upland pine and mixed bottomland hardwood habitats. These bordering areas approximately overlap with the border between poorly drained soil and well-drained soil. In contrast, gravid midges of these two species demonstrate a strong preference for mixed bottomland hardwood habitats with poorly drained soil, expanding out into the mixed hardwood pine habitats, which were avoided by the parous states. Other parts of the ranch with rural/developed/pasture habitats were predicted to be most populated by *C. stellifer* parous midges, with these areas being avoided by *C. stellifer* gravid females, *C. haematopotus* parous females, and *C. haematopotus* gravid females.

**Figure 5:**
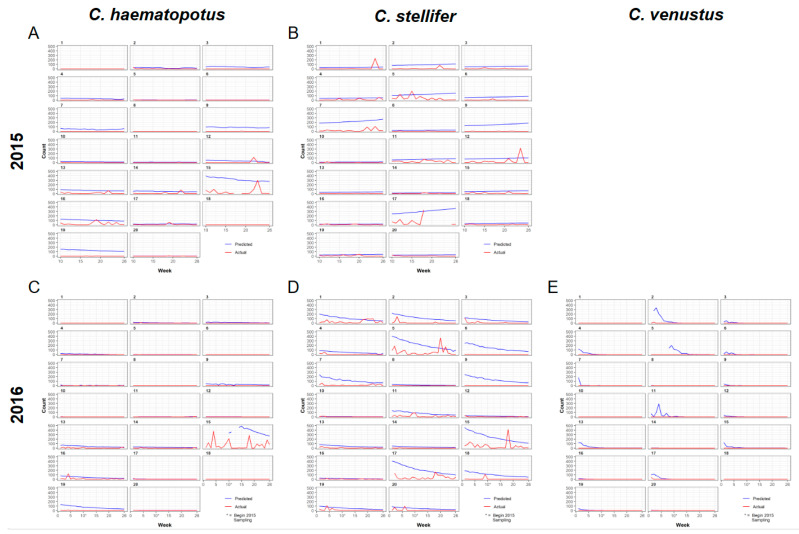
Predicted parous midge counts according to the universal model compared to the actual midge counts observed during the hemorrhagic disease season on a wildlife ranch in the Florida panhandle presented by week and trap number with *C. haematopotus* (**A**) and *C. stellifer* (**B**) data from 2015, and *C. haematopotus* (**C**), *C. stellifer* (**D**), and *C. venustus* (**E**) data from 2016. Counts for *C. venustus* were too low to use for spatial predictions in 2015.

**Figure 6:**
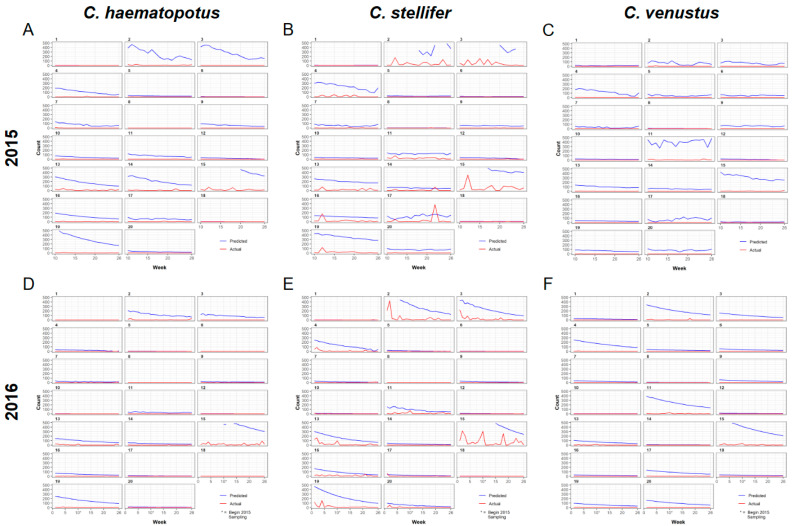
Predicted gravid midge counts according to the universal model compared to the actual midge counts observed during the hemorrhagic disease season on a wildlife ranch in the Florida panhandle presented by week and trap number with *C. haematopotus* (**A**), *C. stellifer* (**B**), and *C. venustus* (**C**) data from 2015, and *C. haematopotus* (**D**), *C. stellifer* (**E**), and *C. venustus* (**F**) data from 2016.

The predicted abundance for parous *C. haematopotus* is comparatively low throughout the season and gradually decreases until week 26 in both years (Figure 5). In contrast, the abundance of parous *C. stellifer* is higher than *C. haematopotus*, and was predicted to slightly increase over the transmission season in 2015 but decrease over the transmission season in 2016 (Figure 5). Abundance of gravid individuals for both species is predicted to be higher and more variable than the abundance of parous individuals, but also gradually decreased throughout the transmission season (Figure 6).

Only 24 samples of parous *C. venustus* were collected in 2015, so spatial predictions for that year and states were not reliable and were excluded. We include predictions for parous *C. venustus* in 2016, when there were 96 individuals. While still a limited sample size compared to *C. haematopotus* and *C. stellifer* in 2016, which had 2403 and 7592 counts, respectively, the predicted abundance of parous *C. venustus* in 2016 appeared to be concentrated along the creek throughout the ranch and quickly decreased by week 14 of the transmission season to have a maximum abundance less than 200 midges throughout the ranch (Figure 5). An abundance of both stages of *C. venustus* is concentrated in the mixed bottomland hardwood habitats with poorly drained soil with a higher predicted abundance for gravid than parous, which also has an overall downward trend over the transmission season (Figure 6).

## 4. Discussion

Hemorrhagic disease, caused by EHDV and BTV, is one of the biggest challenges facing deer ranches across North America. Developing a better understanding of the ecology of the *Culicoides* vectors responsible for transmitting these viruses can help mitigate the threats they pose to deer ranch population management and economic success. The present analysis utilized occupancy modeling to predict the parous and gravid abundance of *C. haematopotus*, *C. stellifer*, and *C. venustus* on a wildlife ranch in the Florida panhandle for the duration of the HD transmission seasons in 2015 and 2016. By evaluating specific ecological criteria of *Culicoides* vectors, our modeling approach developed a universal model to predict spatially explicit counts of suspected vector abundance by species and physiological status for the most abundant species. The universal model allows for comparing species’ abundance and distribution using the same covariates and model structure. These predicted distributions and counts for putative vector species are proxies for HD transmission risk for this landscape. Variation in universal model success led to developing species’ specific models which are provided in the supplement.

We observed similar patterns in actual abundance in our collected samples from 2015 and 2016, with peaks in *C. haematopotus* and *C. stellifer* parous abundance around weeks 17–21, corresponding to late August–mid-September. Previous estimates suggest this is the high point of the EHDV transmission season [3,53], and this abundance peak agrees with previous work on the same ranch, which also reported a peak in EHDV deer mortalities during September [19]. However, the University of Florida Cervidae Health Research Initiative has reported that farmed deer mortalities in the state are generally in late September to mid-October [54], which extends the season beyond the sampling of this study. While sampling may not have included abundance peaks related to these mortalities, *C. stellifer* has previously been linked with EHDV because of its high abundance during outbreaks [55,56] and was corroborated by a study implicating *C. stellifer* and *C. venustus* as vectors of EHDV in Florida [19]. Our data represent a nearly 2.5-fold higher count of *C. stellifer* in 2015 than in 2016. *Culicoides* species are likely to be affected by changes in temperature [21], and on average temperatures in Gadsden County, Florida, were warmer in August 2015 (82.3 °F/27.9 °C) than in August 2016 (81.8 °F/27.7 °C) [57], providing a plausible explanation for the count difference and suggesting that *Culicoides* abundance and the timing of peaks may be more accurately predicted when detailed climate data are incorporated into a predictive model [58]. It may also be that trapping success declines as vector activities shift from night to day as the year progresses [59].

Predicting the timing of peaks in *Culicoides* abundance would be advantageous for developing a vector management plan (such as targeted insecticide use or habitat modification to reduce midge larvae sites) or, more realistically, an animal management plan to avoid areas and times of high vector abundance (such as moving feeders or changing the time of food availability throughout the 24 h period). As a working wildlife ranch, feeder placement and timing of feeding may both be ways to reduce risk. Although we observed similar peaks in our collected samples around weeks 17–21 in both years, the resulting coefficient estimates from our universal model show time as a significant factor in 2016 but not 2015. Similarly, *Culicoides* studies in England found that emergence is continual and highly variable throughout the year [60], suggesting that time in the HD season may not be the most reliable factor for predicting abundance [61]. Here, we show a more accurate predictor of biting midge abundance may be a combination of covariates such as habitat type, proximity to water, proximity to bloodmeals and soil drainage. For example, gravid midge abundance was significantly increased in mixed bottomland hardwood habitats for *C. haematopotus* in 2016 and both *C. stellifer* and *C. venustus* in 2015 and 2016. Over 75% of the mixed bottomland hardwood habitat on the study ranch was classified as having poorly drained soil and gravid midge abundance was also predicted to be significantly increased closer to water for all gravid situations, except for 2015 *C. venustus.* Because gravid midges are most likely to occur near potential oviposition sites, like muddy or sandy substrates on the shores of ponds and streams, that provide the required moisture for larval development [45], it can be reasoned that habitat type, soil type, and boundary areas between well- and poorly drained soil types can be a strong predictor of gravid midge abundance [62].

In contrast, our universal model did not demonstrate a specific habitat preference for parous midges, those that have previously taken a blood meal and, therefore, are potentially infectious. This indicates that parous midges of these three species may be equally adapted to live in upland pine, mixed hardwood pine, or mixed bottomland hardwood habitats. Previous work found that regardless of physiological status, adult *C. stellifer* females preferred habitats in close proximity to supplemental protein feeders [34], yet this preference was not evident when physiological status was factored in, as was also found in the current study. We observed stronger patterns for parous midge habitat use when applying the individual best models for each parous midge cohort investigated. In these models, all parous midge cohorts except for *C. haematopotus* in 2016 were found in areas that were closer to permanent bodies of water, perhaps because they had recently laid eggs in moist environments along water boundaries. Areas immediately surrounding permanent water bodies would also provide parous midges with new blood meals as WTD and other animals will frequent these habitats throughout the day for hydration [63,64].

Our analysis was focused on parous and gravid states because they are most likely to transmit viruses. Covariate estimates reported here demonstrate differences in used habitats between these life stages, indicating the risk of hemorrhagic disease across the study ranch is not random with clear spatial patterns. Our spatial predictions highlight the striking difference in geography between *C. haematopotus* and *C. stellifer,* particularly for the parous life stage. While the covariate estimates do not indicate any species-specific differences, the *C. haematopotus* and *C. stellifer* parous maps from 2015 and 2016 reveal that *C. haematopotus* was most abundant in the northwest corner of the ranch, while *C. stellifer* was most abundant in the southeast corner.

The highest abundance of *C. haematopotus* was congregated closest to trap 5, which was on the bank of the largest permanent body of water, and trap 15, which was close to a frequently used supplemental feeder in mixed bottomland hardwood habitat on the border of well and poorly drained soils. Conversely, *C. stellifer* abundance was greatest surrounding the southern end of the eastern creek on the ranch closest to trap 17, which was on the border of upland pine and rural/developed/pasture habitats and near two supplemental feeders, the nearest of which was in mixed bottomland hardwood habitat. *Culicoides haematopotus* is known to breed in pond margins and the margins of spring-fed streams with freshwater soil habitats [39,45,65]. It is also hypothesized to prefer feeding at tree canopy levels [39,53], and prior bloodmeal analyses have mostly identified birds as host species [38,66], while bloodmeal analyses from *C. stellifer* frequently reveal cervids, particularly white-tailed deer (*Odocoileus virginianus*), as hosts [38,67,68]. Previous work has also shown that, like *C. haematopotus*, *C. stellifer* will occupy the forest canopies for a portion of the day or night, descend for bloodmeals, and then return to the canopy [53]. However, niche partitioning among preferred bloodmeal hosts may have induced analogous partitioning between these species. The environmental features associated with areas of abundance for these two species align well with the likely habitats of the preferred hosts, thus verifying the importance of proximity to bloodmeals for both species.

Collected samples for *C. venustus* were limited for parous midges in both 2015 and 2016, with only 24 and 96 samples, respectively. Count and spatial predictions for 2015 using any model were excluded from this study, and it is likely that the included predictions for 2016 are not reliable. In fact, prior research on the same study ranch found *C. venustus* at traps in the geographic range of *C. haematopotus* [38] rather than in the same area as *C. stellifer*, as shown on the current prediction maps. *Culicoides venustus* gravid counts were also much lower than counts for *C. haematopotus* and *C. stellifer* but were high enough to reasonably model with 331 samples in 2015 and 682 samples in 2016, respectively. Spatial predictions for *C. venustus* were similar for both years but predicted counts were more variable in 2015 than in 2016, suggesting that there may be a specific sample threshold to meet to generate reliable predictive models.

Small sample sizes, such as those for *C. venustus*, are a limitation of this study and affected the breadth of analysis possible. These sample sizes may have been a result of the singular trap height used throughout the entire study, as many species of *Culicoides* frequently move between the ground level and forest canopies throughout the day [53]. However, because WTD is bitten near ground level (not in the canopy), the modeled distributions of midges presented here are relevant. A second limitation is the lack of high-resolution local climate and weather data. Specifically, future studies with this design should deploy wind, temperature, and precipitation measuring devices in proximity to traps to better capture local variation, such as wind or thermal refugia, that may increase catches at some sites. Identifying patterns in those factors would better explain relationships between vector abundance, the timing of vector habitat use, as well as host habitat use, which may better predict midge abundance. Lastly, the continuation of this study through to 2017 informed us that the duration of sampling at the start of this study was not conducted long enough [38]. Because the peak of reported HD mortalities is not reflected in our 2015 or 2016 sampling, it is possible that abundance peaks do not match our modeled estimates [54]. This identifies an area for further study and suggests that year-round trapping may be required to fully determine midge abundance.

## 5. Conclusions

Here, we estimated the abundances of three *Culicoides* species for two physiological states over two years to develop a universal model and better understand the risk of disease transmission during the HD season. Distinct spatial and abundance patterns were identified for both parous and gravid females of two species, *C. haematopotus* and *C. stellifer*. Given the putative vector status of *C. stellifer* for EHDV and lack of evidence for *C. haematopotus* as a putative vector, the differences in habitat use among these two species suggest that the HD risk to deer is not random across the landscape, rather it is correlated to species-specific biting midge abundance and their habitat use. Continued research to further define these patterns can lead to improved animal management strategies that intentionally concentrate animal use in different areas depending on the predicted HD risk.

## Figures and Tables

**Table 1:** Covariates used to model weekly *Culicoides* spp. abundance on a wildlife ranch in the Florida panhandle.

Variable	Description
Week	Week in which samples were collected
Latitude	Insect trap location latitude
Longitude	Insect trap location longitude
Feeder	Euclidian distance from each feeder
Water	Euclidian distance from major water bodies
Habitat	Upland pine, mixed hardwood pine, mixed bottomland Hardwoods, rural/developed/pasture
Soil	Poorly drained, well-drained
Utilization distribution (UD)	Weekly probability density of deer presence based on GPS collar data collected during the study

**Table 2:** Corresponding universal model ΔAICs for each year, *Culicoides* species and state investigated on a wildlife ranch in the Florida panhandle, as well as the individual best model identified for each iteration and the covariates of importance. Gray boxes indicated variables removed from best models.

					Covariates							
Year	Species	Life Stage	Universal Model ΔAIC	Best Model ID	Week	Lat	Long	Hab	Feeder	Water	Soil	UD
2015	*C. haematopotus*	Parous	2.03	G3	X	X	X	X	-	X	X	-
		Gravid	0	GlobalE	X	X	X	X	X	X	X	X
	*C. stellifer*	Parous	2.23	B3	X	X	X	X	X	X	X	-
		Gravid	0.5	B2	X	X	X	X	X	X	X	X
	*C. venustus*	Parous	8.3	H2	X	X	X	-	-	X	X	-
		Gravid	0.88	F1	X	X	X	X	-	X	X	X
2016	*C. haematopotus*	Parous	0	GlobalE	X	X	X	X	X	X	X	X
		Gravid	0	GlobalE	X	X	X	X	X	X	X	X
	*C. stellifer*	Parous	3.543	F4	X	X	X	-	X	X	X	X
		Gravid	0	GlobalE	X	X	X	X	X	X	X	X
	*C. venustus*	Parous	7.17	C2	X	X	X	-	-	X	X	X
		Gravid	1.99	F3	X	X	X	X	X	X	X	-

**Table 3:** Universal models of abundance for three species of *Culicoides* in the parous and gravid states based on sampling during the hemorrhagic disease season in 2015 and 2016. Counts for parous *C. venustus* were too low to use for predictions in 2015.

			2015			2016		
Species	Life Stage	Variable	Estimate	Lower CI	Upper CI	Estimate	Lower CI	Upper CI
*C. haematopotus*	Parous	Intercept	3.9283	1.9899	5.8667	1.3792	0.4080	2.3504
		Week	−0.0251	−0.0976	0.0474	−0.0565	−0.0890	−0.0240
		Latitude	−0.2257	−0.7686	0.3172	−0.7474	−1.1451	−0.3497
		Longitude	1.2017	0.5921	1.8113	1.0429	0.5847	1.5011
		Hardwood Pine	−1.5468	−3.1324	0.0388	0.0905	−1.3615	1.5425
		Mixed Bottomland Hardwoods	−1.1175	−2.3249	0.0899	0.5947	−0.4557	1.6451
		Rural/Developed/Pasture	−3.4974	−4.8400	−2.1548	−2.6068	−4.0055	−1.2081
		Distance to Feeder	−0.2925	−0.8570	0.2720	−0.7749	−1.3125	−0.2373
		Distance to Water	−0.5942	−1.4037	0.2153	−0.7226	−1.4507	0.0055
		Soil—Well-Drained	1.1964	0.1792	2.2136	1.7574	0.9146	2.6002
		UD	−0.2319	−0.6396	0.1758	−0.8701	−1.4940	−0.2462
	Gravid	Intercept	4.7592	3.7126	5.8058	1.8442	0.9157	2.7727
		Week	−0.0728	−0.1134	−0.0322	−0.0422	−0.0692	−0.0152
		Latitude	0.1728	−0.1518	0.4974	−0.2125	−0.5377	0.1127
		Longitude	0.4277	0.0402	0.8152	0.5066	0.0411	0.9721
		Hardwood Pine	0.7949	−0.3094	1.8992	0.7308	−0.5557	2.0173
		Mixed Bottomland Hardwoods	0.6951	−0.0179	1.4081	1.5035	0.6950	2.3120
		Rural/Developed/Pasture	−1.9882	−2.9521	−1.0243	−2.8471	−4.6562	−1.0380
		Distance to Feeder	−0.6294	−1.0098	−0.2490	−0.8668	−1.3474	−0.3862
		Distance to Water	−0.6552	−1.2097	−0.1007	−1.2174	−1.9851	−0.4497
		Soil—Well-Drained	0.7038	0.0339	1.3737	1.0071	0.3672	1.6470
		UD	−0.2136	−0.4911	0.0639	−0.3294	−0.8327	0.1739
*C. stellifer*	Parous	Intercept	4.221	3.2388	5.2032	3.9575	3.3369	4.5780
		Week	0.0238	−0.0156	0.0632	−0.0577	−0.0787	−0.0367
		Latitude	0.153	−0.1383	0.4443	−0.0024	−0.2684	0.2636
		Longitude	−0.3199	−0.5865	−0.0533	−0.4059	−0.6548	−0.1569
		Hardwood Pine	−1.7376	−2.6582	−0.8170	0.0597	−0.8493	0.9688
		Mixed Bottomland Hardwoods	−1.2134	−1.8455	−0.5813	0.2423	−0.3174	0.8021
		Rural/Developed/Pasture	−0.8437	−1.4811	−0.2063	0.4621	−0.1151	1.0393
		Distance to Feeder	−0.2166	−0.4673	0.0341	−0.7959	−1.0442	−0.5475
		Distance to Water	−0.6428	−0.9499	−0.3357	−0.7113	−0.9806	−0.4420
		Soil—Well-Drained	0.4181	−0.2209	1.0571	1.0961	0.5663	1.6259
		UD	−0.0101	−0.2290	0.2088	−0.1003	−0.3035	0.1030
	Gravid	Intercept	4.1836	3.1201	5.2471	2.5868	2.0634	3.1102
		Week	−0.0293	−0.0734	0.0148	−0.0650	−0.0832	−0.0468
		Latitude	0.5101	0.2461	0.7741	0.0947	−0.0945	0.2839
		Longitude	0.0741	−0.1995	0.3477	0.2074	−0.0086	0.4234
		Hardwood Pine	−0.1702	−1.1055	0.7651	0.5949	−0.2403	1.4301
		Mixed Bottomland Hardwoods	1.3422	0.6578	2.0266	2.5083	2.0242	2.9924
		Rural/Developed/Pasture	−1.6848	−2.4184	−0.9512	−0.9730	−1.5591	−0.3869
		Distance to Feeder	−0.6471	−0.9144	−0.3798	−0.6411	−0.8746	−0.4076
		Distance to Water	−0.3603	−0.7431	0.0225	−0.6112	−0.9108	−0.3116
		Soil—Well-Drained	0.4308	−0.2076	1.0692	0.8486	0.4276	1.2696
		UD	−0.3187	−0.5208	−0.1166	−0.1660	−0.3421	0.0101
*C. venustus*	Parous	Intercept				3.2275	0.0268	6.4282
		Week				−0.4112	−0.6425	−0.1799
		Latitude				−0.2923	−1.7231	1.1385
		Longitude				−1.2482	−3.1435	0.6471
		Hardwood Pine				−7.9021	−75.5848	59.7806
		Mixed Bottomland Hardwoods				−0.3547	−3.5319	2.8225
		Rural/Developed/Pasture				0.0806	−3.9727	4.1339
		Distance to Feeder				0.6702	−1.1683	2.5087
		Distance to Water				−3.6087	−6.6193	−0.5981
		Soil—Well-Drained				1.1208	−1.3998	3.6414
		UD				−1.9747	−3.9974	0.0480
	Gravid	Intercept	3.1712	1.6934	4.6490	2.3449	1.3514	3.3384
		Week	−0.0375	−0.0794	0.0044	−0.0435	−0.0696	−0.0174
		Latitude	−0.2011	−0.4847	0.0825	0.0413	−0.1767	0.2593
		Longitude	−0.8125	−1.1620	−0.4630	−0.6835	−0.9936	−0.3734
		Hardwood Pine	1.6433	0.4056	2.8810	0.0416	−1.6707	1.7539
		Mixed Bottomland Hardwoods	1.4783	0.7313	2.2253	2.3591	1.7362	2.9820
		Rural/Developed/Pasture	−0.4192	−1.2796	0.4412	−0.0916	−0.8611	0.6779
		Distance to Feeder	−0.1861	−0.5326	0.1604	−0.5462	−0.8476	−0.2448
		Distance to Water	−1.1374	−1.5835	−0.6913	−0.8012	−1.1938	−0.4086
		Soil—Well-Drained	1.1702	0.5363	1.8041	1.4401	0.9021	1.9781
		UD	−0.5041	−0.8694	−0.1388	−0.0125	−0.2385	0.2135

## Data Availability

The datasets and R code generated and/or analyzed during the current study are not publicly available due to private landowner privacy agreements but are available from the corresponding author upon reasonable request.

## Supplementary Materials

The following supporting information can be downloaded at: https://www.mdpi.com/article/10.3390/v16050766/s1, Table S1. Pearson correlation r values for assessing correlation between continuous covariates used to model *Culicoides* spp. weekly abundance on a wildlife ranch in the Florida panhandle. Table S2. ANOVA *p*-values for testing correlation between continuous numerical and categorical variables used to model *Culicoides* spp. weekly abundance on a wildlife ranch in the Florida panhandle. Table S3. List of models that were run to compare how selected variables relate to *Culicoides* midge preferences by species, physiological state, and year. Table S4. Summary of midge samples identified to species and physiological state that were collected from a wildlife ranch in the Florida panhandle between July–October 2015 and May–October 2016. Table S5. Individual best models of abundance for three species of *Culicoides* in the parous and gravid physiological states based on sampling during the hemorrhagic disease (HD) season in 2015 and 2016. Covariate estimates with blank cells were not included in the selected individual best model for the specific year, species, and physiological state, and estimates with shaded cells are identical to those reported in Table S5. Counts for parous *C. venustus* were too low to use for predictions in 2015. Figure S1. The total number of *Culicoides* midges collected and identified by species during the 2015 (A) and 2016 (B) sampling seasons on a wildlife ranch in the Florida panhandle. Figure S2. Still image of GIFs included in the supplemental PowerPoint file illustrating the predicted parous and gravid *Culicoides* abundance on a wildlife ranch in the Florida panhandle during the 2015 hemorrhagic disease transmission season using the universal model to make predictions for parous *C. haematopotus* (A) and *C. stellifer* (B), and gravid *C. haematopotus* (C), *C. stellifer* (D), and *C. venustus* (E). Figure S3. Still image of GIFs included in the supplemental PowerPoint file illustrating the predicted parous and gravid *Culicoides* abundance on a wildlife ranch in the Florida panhandle during the 2016 hemorrhagic disease transmission season using the universal model to make predictions for parous *C. haematopotus* (A), *C. stellifer* (B), and *C. venustus* (C), and gravid *C. haematopotus* (D), *C. stellifer* (E), and *C. venustus* (F). Figure S4. Predicted parous *Culicoides* abundance on a wildlife ranch in the Florida panhandle during the 14th week (8 March 2015–8 July 2015, 8 January 2016–8 May 2016) of the sampling season using the individual best model for each year, species, and physiological state to make predictions for *C. haematopotus* (A) and *C. stellifer* (B) in 2015, and predictions for *C. haematopotus* (C), *C. stellifer* (D), and *C. venustus* (E) in 2016. Counts for *C. venustus* were too low to use for spatial predictions in 2015. Figure S5. Predicted gravid *Culicoides* abundance on a deer ranch in the Florida panhandle during the 14th week (8 March 2015–8 July 2015, 8 January 2016–8 May 2016) of the HD transmission season using the individual best model for each year, species and physiological state to make predictions for *C. haematopotus* (A), *C. stellifer* (B), and *C. venustus* (C) in 2015, and predictions for *C. haematopotus* (D), *C. stellifer* (E), and *C. venustus* (F) in 2016. Figure S6. Still image of GIFs included in the supplemental PowerPoint file illustrating the predicted parous and gravid *Culicoides* abundance on a wildlife ranch in the Florida panhandle during the 2015 hemorrhagic disease transmission season. Maps were created using the individual best model for each year, species, and physiological state to make predictions for parous *C. haematopotus* (A) and *C. stellifer* (B), and gravid *C. haematopotus* (C), *C. stellifer* (D), and *C. venustus* (E). Best model IDs are noted in each panel and correspond to Tables S4 and S1, with covariate estimates displayed in Table S5. Figure S7. Still image of GIFs included in the supplemental PowerPoint file illustrating the predicted parous and gravid *Culicoides* abundance on a wildlife ranch in the Florida panhandle during the 2016 hemorrhagic disease transmission season. Maps were created using the individual best model for each year, species, and physiological state to make predictions for parous *C. haematopotus* (A), *C. stellifer* (B), and *C. venustus* (C), and gravid *C. haematopotus* (D), *C. stellifer* (E), and *C. venustus* (F). Best model IDs are noted in each panel and correspond to Tables S4 and S1, with covariate estimates displayed in Table S5. Figure S8. Predicted parous midge counts according to the individual best model compared to the actual midge counts observed during the hemorrhagic disease season on a wildlife ranch in the Florida panhandle presented by week and trap number with *C. haematopotus* (A) and *C. stellifer* (B) data from 2015, and *C. haematopotus* (C), *C. stellifer* (D), and *C. venustus* (E) data from 2016. Counts for *C. venustus* were too low to use for spatial predictions in 2015. Figure S9. Predicted gravid midge counts according to the individual best model compared to the actual midge counts observed during the hemorrhagic disease season on a wildlife ranch in the Florida panhandle presented by week and trap number with *C. haematopotus* (A), *C. stellifer* (B), and *C. venustus* (C) data from 2015, and *C. haematopotus* (D), *C. stellifer* (E), and *C. venustus* (F) data from 2016.

## References

[1] Stevens G., McCluskey B., King A., O’Hearn E., Mayr G. (2015). Review of the 2012 Epizootic Hemorrhagic Disease Outbreak in Domestic Ruminants in the United States. PLoS ONE.

[2] Wilson A., Mellor P. (2008). Bluetongue in Europe: Vectors, Epidemiology and Climate Change. Parasitol. Res..

[3] Ruder M.G., Lysyk T.J., Stallknecht D.E., Foil L.D., Johnson D.J., Chase C.C., Dargatz D.A., Gibbs E.P.J. (2015). Transmission and Epidemiology of Bluetongue and Epizootic Hemorrhagic Disease in North America: Current Perspectives, Research Gaps, and Future Directions. Vector-Borne Zoonotic Dis..

[4] Savini G., Afonso A., Mellor P., Aradaib I., Yadin H., Sanaa M., Wilson W., Monaco F., Domingo M. (2011). Epizootic Haemorragic Disease. Res. Vet. Sci..

[5] Stair E.L., Robinson R.M., Jones L.P. (1968). Spontaneous Bluetongue in Texas White-Tailed Deer. Pathol. Vet..

[6] Howerth E.W., Stallknecht D.E., Kirkland Peter D., Williams E.S., Barker I.K. (2000). Bluetongue, epizootic hemorrhagic disease, and other orbivirus-related diseases. Infectious Diseases of Wild Mammals.

[7] Nol P., Kato C., Reeves W.K., Rhyan J., Spraker T., Gidlewski T., VerCauteren K., Salman M. (2010). Epizootic Hemorrhagic Disease Outbreak in a Captive Facility Housing White-Tailed Deer (*Odocoileus virginianus*), Bison (*Bison bison*), Elk (*Cervus elaphus*), Cattle (*Bos taurus*), and Goats (*Capra hircus*) in Colorado, U.S.A. J. Zoo Wildl. Med..

[8] Thorne E.T., Williams E.S., Spraker T.R., Helms W., Segerstrom T. (1988). Bluetongue in free-ranging pronghorn antelope (antilocapra americana) in wyoming: 1976 and 1984. JWDI.

[9] Dubay S.A., Noon T.H., deVos J.C., Ockenfels R.A. (2006). Serologic Survey for Pathogens Potentially Affecting Pronghorn (Antilocapra Americana) Fawn Recruitment in Arizona, USA. JWDI.

[10] Robinson R.M., Hailey T.L., Livingston C.W., Thomas J.W. (1967). Bluetongue in the Desert Bighorn Sheep. J. Wildl. Manag..

[11] Ruder M.G., Allison A.B., Stallknecht D.E., Mead D.G., McGraw S.M., Carter D.L., Kubiski S.V., Batten C.A., Klement E., Howerth E.W. (2012). Susceptibility of white-tailed deer (*Odocoileus virginianus*) to experimental infection with epizootic hemorrhagic disease virus serotype 7. J. Wildl. Dis..

[12] Allison A.B., Goekjian V.H., Potgieter A.C., Wilson W.C., Johnson D.J., Mertens P.P.C., Stallknecht D.E. (2010). Detection of a Novel Reassortant Epizootic Hemorrhagic Disease Virus (EHDV) in the USA Containing RNA Segments Derived from Both Exotic (EHDV-6) and Endemic (EHDV-2) Serotypes. J. Gen. Virol..

[13] Johnson D.J., Ostlund E.N., Stallknecht D.E., Goekjian V.H., Jenkins-Moore M., Harris S.C. (2006). First Report of Bluetongue Virus Serotype 1 Isolated from a White-Tailed Deer in the United States. J. Vet. Diagn. Investig..

[14] Drolet B.S., Reister L.M., Rigg T.D., Nol P., Podell B.K., Mecham J.O., VerCauteren K.C., van Rijn P.A., Wilson W.C., Bowen R.A. (2013). Experimental Infection of White-Tailed Deer (*Odocoileus virginianus*) with Northern European Bluetongue Virus Serotype 8. Vet. Microbiol..

[15] Gaydos J.K., Crum J.M., Davidson W.R., Cross S.S., Owen S.F., Stallknecht D.E. (2004). Epizootiology of an epizootic hemorrhagic disease outbreak in West Virginia. JWDI.

[16] Anderson D.P., Frosch B.J., Outlaw J.L. (2007). Economic Impact of the United States Cervid Farming Industry.

[17] Anderson D.P., Outlaw J.L., Earle M.L., Richardson J.W. (2017). Economic Impact of Texas Deer Breeding and Hunting Operations.

[18] Stallknecht D.E., Luttrell M.P., Smith K.E., Nettles V.F. (1996). Hemorrhagic Disease in White-Tailed Deer in Texas: A Case for Enzootic Stability. JWDI.

[19] McGregor B.L., Sloyer K.E., Sayler K.A., Goodfriend O., Krauer J.M.C., Acevedo C., Zhang X., Mathias D., Wisely S.M., Burkett-Cadena N.D. (2019). Field Data Implicating Culicoides Stellifer and Culicoides Venustus (Diptera: Ceratopogonidae) as Vectors of Epizootic Hemorrhagic Disease Virus. Parasites Vectors.

[20] Cottingham S.L., White Z.S., Wisely S.M., Campos-Krauer J.M. (2021). A Mortality-Based Description of EHDV and BTV Prevalence in Farmed White-Tailed Deer (*Odocoileus virginianus*) in Florida, USA. Viruses.

[21] Purse B.V., Mellor P.S., Rogers D.J., Samuel A.R., Mertens P.P.C., Baylis M. (2005). Climate Change and the Recent Emergence of Bluetongue in Europe. Nat. Rev. Microbiol..

[22] Noronha L.E., Cohnstaedt L.W., Richt J.A., Wilson W.C. (2021). Perspectives on the Changing Landscape of Epizootic Hemorrhagic Disease Virus Control. Viruses.

[23] Allen S.E., Rothenburger J.L., Jardine C.M., Ambagala A., Hooper-McGrevy K., Colucci N., Furukawa-Stoffer T., Vigil S., Ruder M., Nemeth N.M. (2019). Epizootic Hemorrhagic Disease in White-Tailed Deer, Canada. Emerg. Infect. Dis..

[24] Allen S.E., Jardine C.M., Hooper-McGrevy K., Ambagala A., Bosco-Lauth A.M., Kunkel M.R., Mead D.G., Nituch L., Ruder M.G., Nemeth N.M. (2020). Serologic Evidence of Arthropod-Borne Virus Infections in Wild and Captive Ruminants in Ontario, Canada. Am. J. Trop. Med. Hyg..

[25] Allison A.B., Holmes E.C., Potgieter A.C., Wright I.M., Sailleau C., Breard E., Ruder M.G., Stallknecht D.E. (2012). Segmental Configuration and Putative Origin of the *Reassortant orbivirus*, Epizootic Hemorrhagic Disease Virus Serotype 6, Strain Indiana. Virology.

[26] Carpenter S., McArthur C., Selby R., Ward R., Nolan D.V., Luntz A.J.M., Dallas J.F., Tripet F., Mellor P.S. (2008). Experimental Infection Studies of UK *Culicoides* Species Midges with Bluetongue Virus Serotypes 8 and 9. Vet. Rec..

[27] Elbers A.R.W., Backx A., Meroc E., Gerbier G., Staubach C., Hendrickx G., van der Spek A., Mintiens K. (2008). Field Observations during the Bluetongue Serotype 8 Epidemic in 2006: I. Detection of First Outbreaks and Clinical Signs in Sheep and Cattle in Belgium, France and the Netherlands. Prev. Vet. Med..

[28] Maan S., Maan N.S., Ross-smith N., Batten C.A., Shaw A.E., Anthony S.J., Samuel A.R., Darpel K.E., Veronesi E., Oura C.A.L. (2008). Sequence Analysis of Bluetongue Virus Serotype 8 from the Netherlands 2006 and Comparison to Other European Strains. Virology.

[29] Maan S., Maan N.S., Rijn P.A.V., van Gennip R.G., Sanders A., Wright I.M., Batten C., Hoffmann B., Eschbaumer M., Oura C.A.L. (2010). Full Genome Characterisation of Bluetongue Virus Serotype 6 from the Netherlands 2008 and Comparison to Other Field and Vaccine Strains. PLoS ONE.

[30] Elbers A.R.W., Koenraadt C., Meiswinkel R. (2015). Mosquitoes and Culicoides Biting Midges: Vector Range and the Influence of Climate Change: -EN- -FR-Les Moustiques et Les Moucherons Piqueurs Culicoides: Diversité Des Vecteurs et Influence Du Changement Climatique-ES-Mosquitos y Jejenes Culicoides: Distribución de Los Vectores e Influencia Del Cambio Climático. Rev. Sci. Tech. OIE.

[31] Foxi C., Delrio G., Falchi G., Marche M.G., Satta G., Ruiu L. (2016). Role of Different Culicoides Vectors (Diptera: Ceratopogonidae) in Bluetongue Virus Transmission and Overwintering in Sardinia (Italy). Parasites Vectors.

[32] Calvo J.H., Berzal B., Calvete C., Miranda M.A., Estrada R., Lucientes J. (2012). Host Feeding Patterns of *Culicoides* Species (Diptera: Ceratopogonidae) within the Picos de Europa National Park in Northern Spain. Bull. Entomol. Res..

[33] Vasić A., Zdravković N., Aniță D., Bojkovski J., Marinov M., Mathis A., Niculaua M., Oșlobanu E.L., Pavlović I., Petrić D. (2019). Species Diversity, Host Preference and Arbovirus Detection of Culicoides (Diptera: Ceratopogonidae) in South-Eastern Serbia. Parasites Vectors.

[34] Dinh E.T.N., Gomez J.P., Orange J.P., Morris M.A., Sayler K.A., McGregor B.L., Blosser E.M., Burkett-Cadena N.D., Wisely S.M., Blackburn J.K. (2021). Modeling Abundance of Culicoides Stellifer, a Candidate Orbivirus Vector, Indicates Nonrandom Hemorrhagic Disease Risk for White-Tailed Deer (*Odocoileus virginianus*). Viruses.

[35] Purse B.V., Carpenter S., Venter G.J., Bellis G., Mullens B.A. (2015). Bionomics of Temperate and Tropical Culicoides Midges: Knowledge Gaps and Consequences for Transmission of Culicoides-Borne Viruses. Annu. Rev. Entomol..

[36] Carpenter S., Wilson A., Mellor P.S. (2009). Culicoides and the Emergence of Bluetongue Virus in Northern Europe. Trends Microbiol..

[37] Cauvin A., Dinh E.T.N., Orange J.P., Shuman R.M., Blackburn J.K., Wisely S.M. (2020). Antibodies to Epizootic Hemorrhagic Disease Virus (EHDV) in Farmed and Wild Florida White-Tailed Deer (*Odocoileus virginianus*). J. Wildl. Dis..

[38] McGregor B.L., Stenn T., Sayler K.A., Blosser E.M., Blackburn J.K., Wisely S.M., Burkett-Cadena N.D. (2019). Host Use Patterns of *Culicoides* Spp. Biting Midges at a Big Game Preserve in Florida, U.S.A., and Implications for the Transmission of Orbiviruses. Med. Vet. Entomol..

[39] Blanton F.S., Wirth W.W. (1979). The Sand Flies (Culicoides) of Florida (Diptera: Ceratopogonidae). Arthropods of Florida and Neighboring Land Areas.

[40] Holbrook F.R., Beaty B.J., Marquardt W.C. (1996). Biting MIdges And The Agents They Transmit. The Biology of Disease Vectors.

[41] Cooperative Land Cover, Version 3.5—Published November 2021. https://myfwc.com/research/gis/regional-projects/cooperative-land-cover/.

[42] Dinh E.T.N. (2019). Epizootic Hemorrhagic Disease Virus Transmission Risk in North Florida Free-Ranging Ranched and Wild White-Tailed Deer. Ph.D. Thesis.

[43] Web Soil Survey—Home. https://websoilsurvey.sc.egov.usda.gov/App/HomePage.htm.

[44] Erram D., Burkett-Cadena N. (2018). Laboratory Studies on the Oviposition Stimuli of Culicoides Stellifer (Diptera: Ceratopogonidae), a Suspected Vector of Orbiviruses in the United States. Parasites Vectors.

[45] Erram D., Blosser E.M., Burkett-Cadena N. (2019). Habitat Associations of Culicoides Species (Diptera: Ceratopogonidae) Abundant on a Commercial Cervid Farm in Florida, USA. Parasites Vectors.

[46] Worton B.J. (1989). Kernel Methods for Estimating the Utilization Distribution in Home-Range Studies. Ecology.

[47] Getz W.M., Wilmers C.C. (2004). A Local Nearest-Neighbor Convex-Hull Construction of Home Ranges and Utilization Distributions. Ecography.

[48] Dinh E.T.N., Cauvin A., Orange J.P., Shuman R.M., Wisely S.M., Blackburn J.K. (2020). Living La Vida T-LoCoH: Site Fidelity of Florida Ranched and Wild White-Tailed Deer (*Odocoileus virginianus*) during the Epizootic Hemorrhagic Disease Virus (EHDV) Transmission Period. Mov. Ecol..

[49] Fiske I., Chandler R. (2011). Unmarked: An R Package for Fitting Hierarchical Models of Wildlife Occurrence and Abundance. J. Stat. Softw..

[50] Fiske I., Chandler R. Overview of Unmarked: An R Package for the Analysis of Data from Unmarked Animals. https://citeseerx.ist.psu.edu/document?repid=rep1&type=pdf&doi=a6120f7b6305fdd39c429c8738f2eec24196b2be.

[51] A Method for Estimating Insect Abundance and Patch Occupancy with Potential Applications in Large-Scale Monitoring Programmes. https://journals.co.za/doi/10.10520/EJC32718.

[52] Royle J.A. (2004). N-Mixture Models for Estimating Population Size from Spatially Replicated Counts. Biometrics.

[53] McGregor B.L., Runkel A.E., Wisely S.M., Burkett-Cadena N.D. (2018). Vertical Stratification of Culicoides Biting Midges at a Florida Big Game Preserve. Parasites Vectors.

[54] CHeRI HD Cases: Dashboard Experience. https://experience.arcgis.com/experience/3a08f867439043b1a71e280f73495037/.

[55] Smith K.E., Stallknecht D.E. (1996). Culicoides (Diptera: Ceratopogonidae) Collected During Epizootics of Hemorrhagic Disease among Captive White-Tailed Deer. J. Med. Entomol..

[56] Smith K.E., Stallknecht D.E., Sewell C.T., Iii E.A.R., Mullen G.R., Anderson R.R. (1996). Monitoring of *Culicoides* Spp. at a site enzootic for hemorrhagic disease in white-tailed deer in Georgia, USA. JWDI.

[57] Climate at a Glance|National Centers for Environmental Information (NCEI). https://www.ncei.noaa.gov/access/monitoring/climate-at-a-glance/county/rankings/FL-039/tavg/201608.

[58] Sanders C.J., Shortall C.R., England M., Harrington R., Purse B., Burgin L., Carpenter S., Gubbins S. (2019). Long-Term Shifts in the Seasonal Abundance of Adult Culicoides Biting Midges and Their Impact on Potential Arbovirus Outbreaks. J. Appl. Ecol..

[59] West R.G., Mathias D.R., Day J.F., Boohene C.K., Unnasch T.R., Burkett-Cadena N.D. (2020). Vectorial Capacity of Culiseta Melanura (Diptera: Culicidae) Changes Seasonally and Is Related to Epizootic Transmission of Eastern Equine Encephalitis Virus in Central Florida. Front. Ecol. Evol..

[60] Stokes J.E., Carpenter S., Sanders C., Gubbins S. (2022). Emergence Dynamics of Adult Culicoides Biting Midges at Two Farms in South-East England. Parasites Vectors.

[61] Sayler K., Blosser E., McGregor B., Burkett-Cadena N., Wisely S.M. (2016). Overwintering of Epizootic Hemorrhagic Disease Virus in White-Tailed Deer in Florida, USA: Unanticipated Seroconversion and the Case for Alternative Vectors. Int. J. Infect. Dis..

[62] Schmidtmann E.T., Bobian R.J., Belden R.P. (2000). Soil Chemistries Define Aquatic Habitats with Immature Populations of the Culicoides Variipennis Complex (Diptera: Ceratopogonidae). J. Med. Entomol..

[63] Mandujano S., Gallina S., JAS S.-R., Arceo G., Silva-Villilobos G. (1997). Habitat Use by White-Tailed Deer in a Tropical Forest.

[64] Dryden G. (2011). McL. Quantitative Nutrition of Deer: Energy, Protein and Water. Anim. Prod. Sci..

[65] Atchley W.R., Wirth W.W. (1979). A Review of the Culicoides Haematopotus Group in North America (Diptera: Ceratopogonidae). J. Kans. Entomol. Soc..

[66] Swanson D.A., Turnbull M.W. (2014). Molecular Identification of Bloodmeals from Culicoides Latreille (Diptera: Ceratopogonidae) in the Southeastern U.S.A. Proc. Entomol. Soc. Wash..

[67] McGregor B.L., Blackburn J.K., Wisely S.M., Burkett-Cadena N.D. (2021). Culicoides (Diptera: Ceratopogonidae) Communities Differ Between a Game Preserve and Nearby Natural Areas in Northern Florida. J. Med. Entomol..

[68] Hopken M.W., Ryan B.M., Huyvaert K.P., Piaggio A.J. (2017). Picky Eaters Are Rare: DNA-Based Blood Meal Analysis of Culicoides (Diptera: Ceratopogonidae) Species from the United States. Parasites Vectors.

