# Supplementary material for: Culicoides Midge Abundance across Years: Modeling Inter-Annual Variation for an Avian Feeder and a Candidate Vector of Hemorrhagic Diseases in Farmed Wildlife
Source: Viruses. 2024 May 11;16(5):766. doi: 10.3390/v16050766 (PMC11125994; doi:10.3390/v16050766)
Supplement: Supplementary file 1 [file viruses-16-00766-s001.zip › SupplementaryGIFs.pptx]

## Slide 1
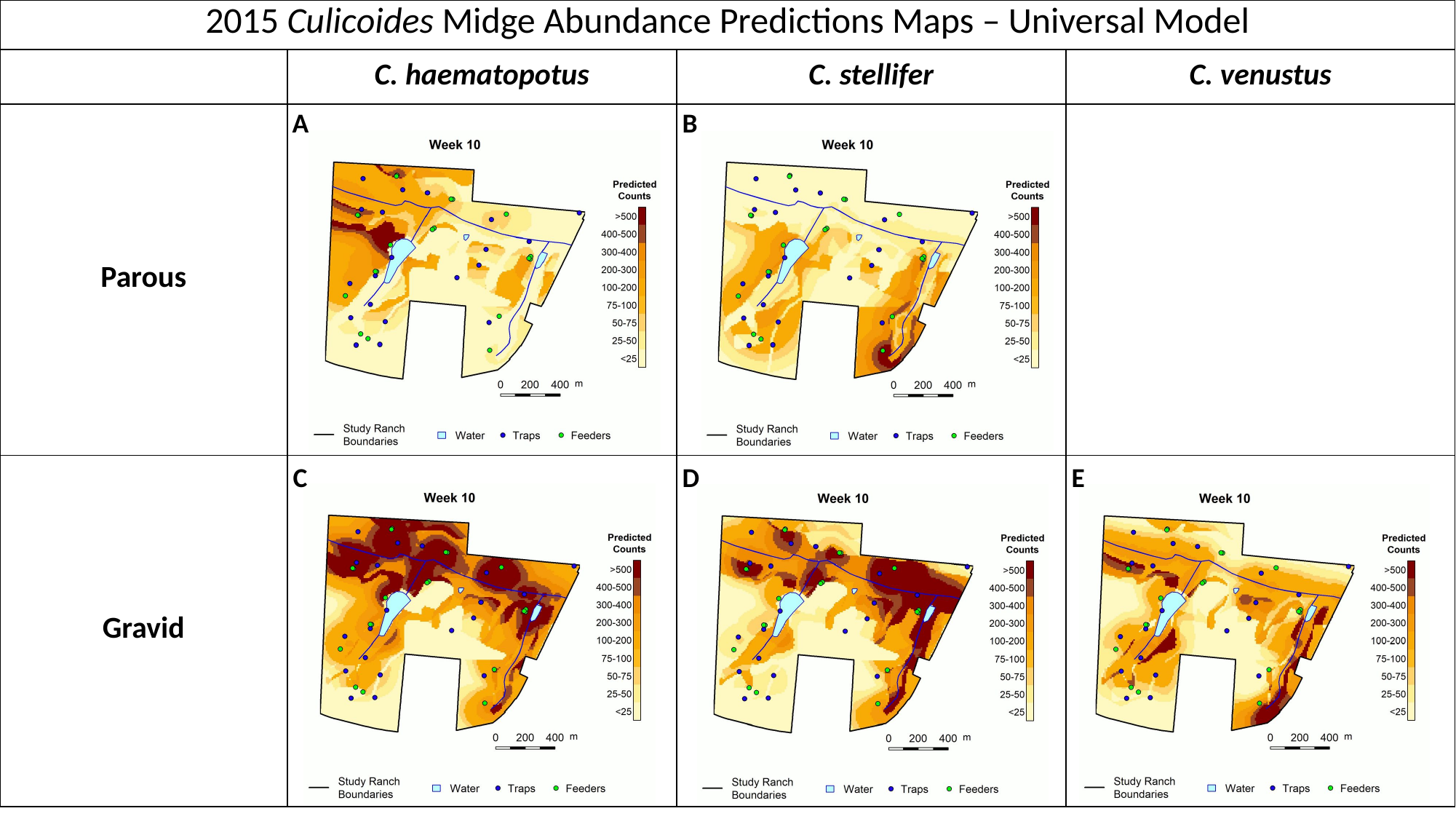

| 2015 Culicoides Midge Abundance Predictions Maps – Universal Model | | | |
| --- | --- | --- | --- |
| | C. haematopotus | C. stellifer | C. venustus |
| Parous | | | |
| Gravid | | | |
A
B
C
D
E

## Slide 2
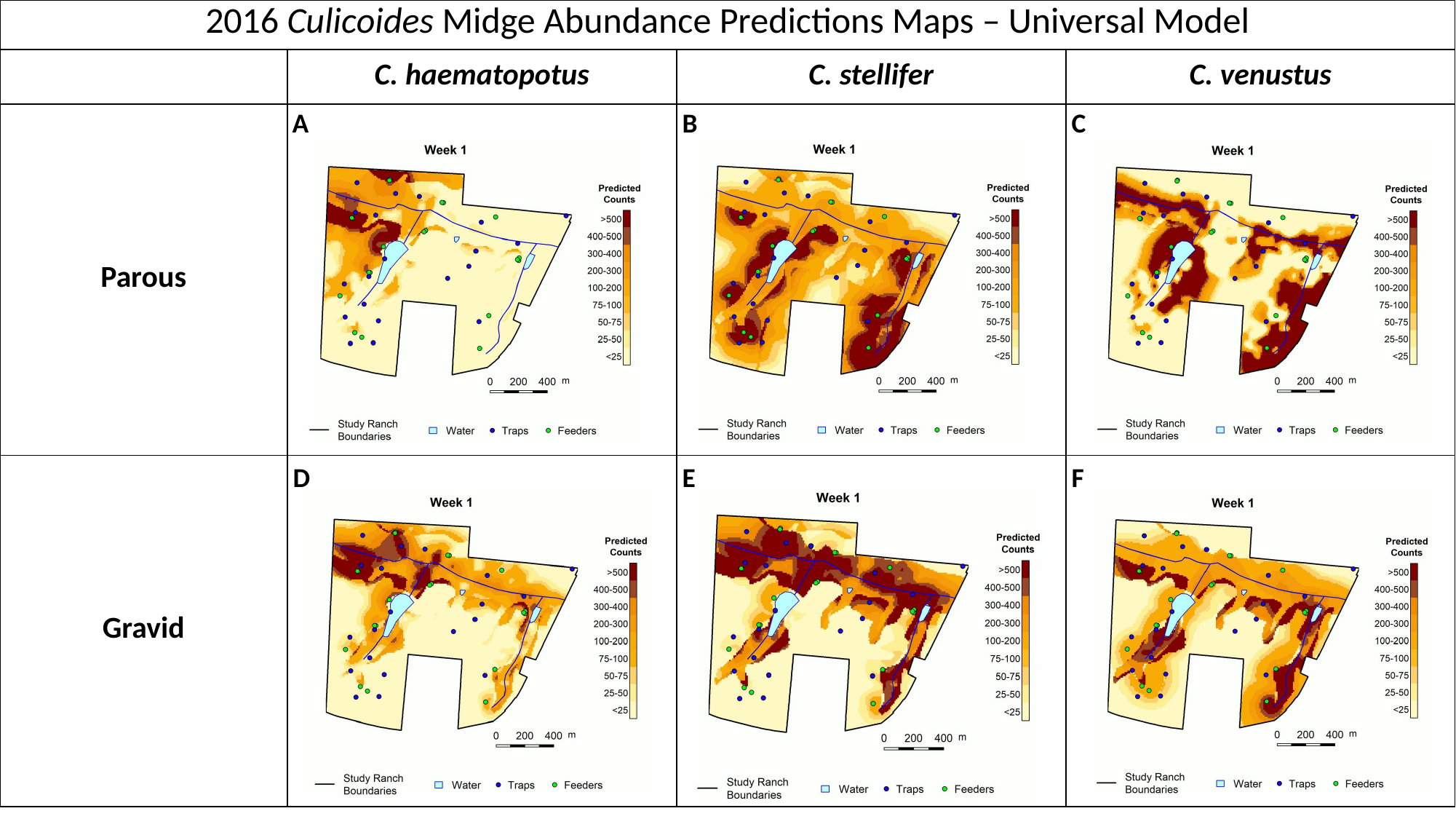

| 2016 Culicoides Midge Abundance Predictions Maps – Universal Model | | | |
| --- | --- | --- | --- |
| | C. haematopotus | C. stellifer | C. venustus |
| Parous | | | |
| Gravid | | | |
A
B
C
D
E
F

## Slide 3
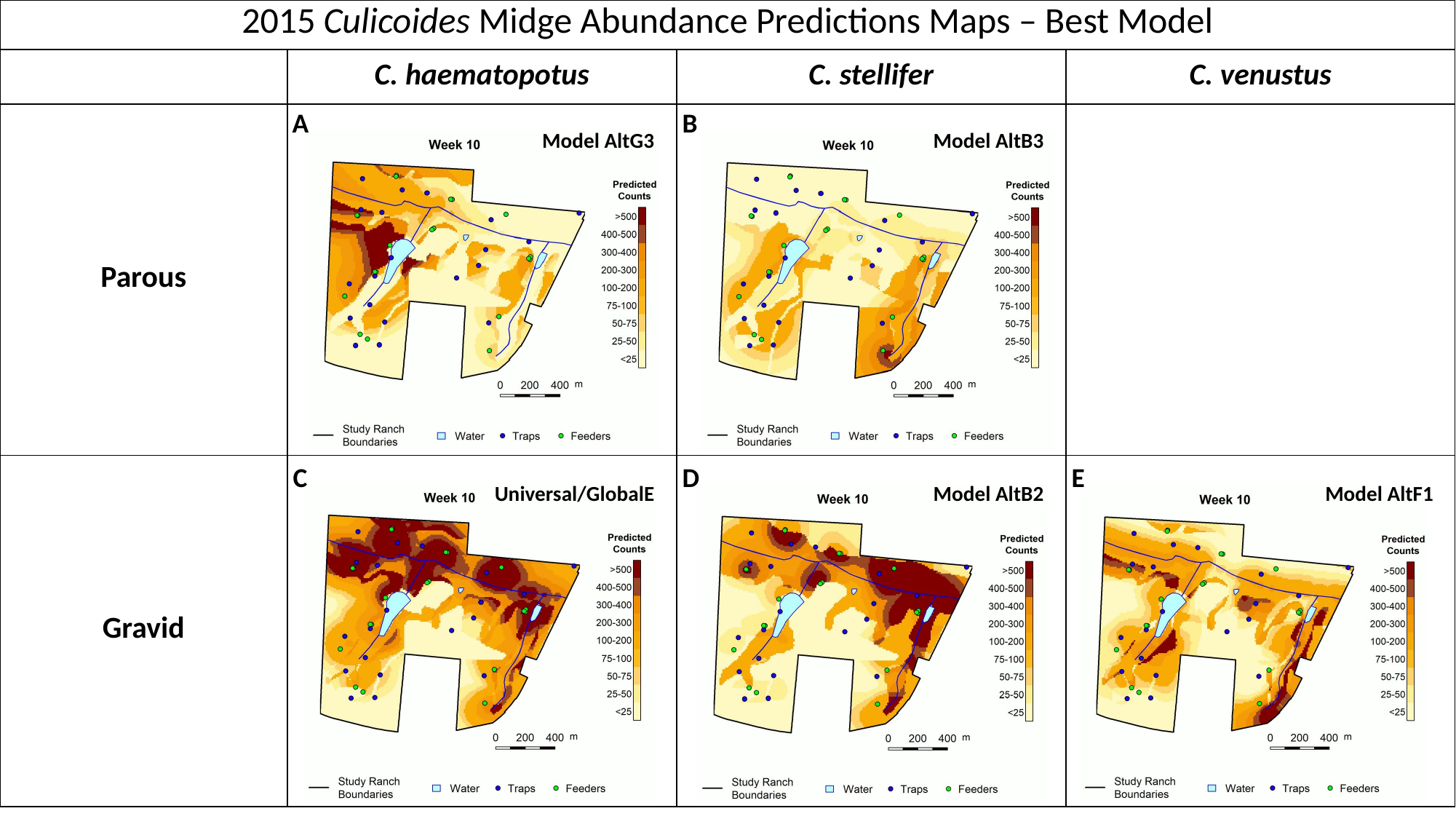

| 2015 Culicoides Midge Abundance Predictions Maps – Best Model | | | |
| --- | --- | --- | --- |
| | C. haematopotus | C. stellifer | C. venustus |
| Parous | | | |
| Gravid | | | |
A
B
C
D
E
Model AltG3
Model AltB3
Model AltF1
Universal/GlobalE
Model AltB2

## Slide 4
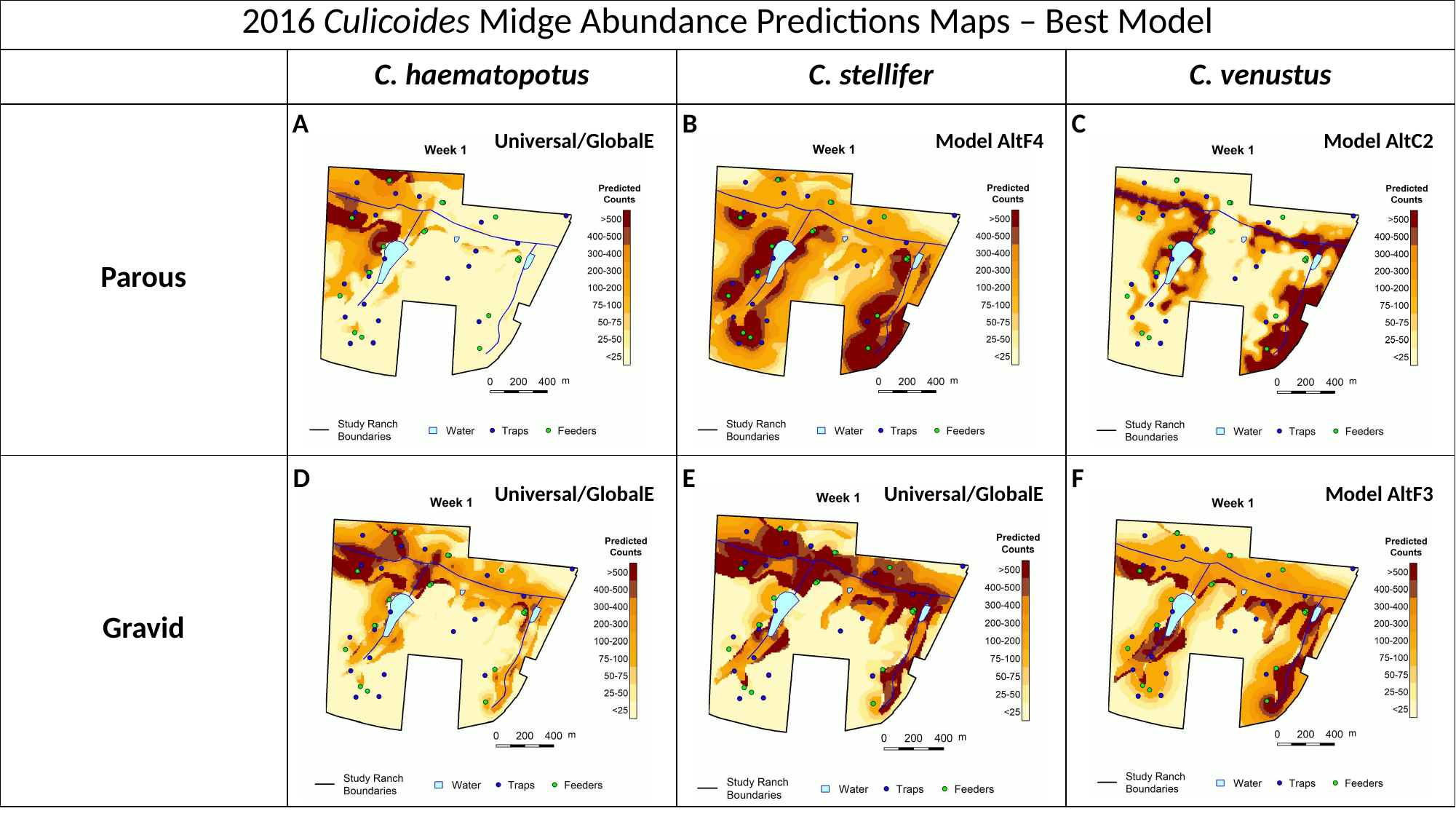

| 2016 Culicoides Midge Abundance Predictions Maps – Best Model | | | |
| --- | --- | --- | --- |
| | C. haematopotus | C. stellifer | C. venustus |
| Parous | | | |
| Gravid | | | |
A
B
C
D
E
F
Universal/GlobalE
Model AltF4
Model AltC2
Model AltF3
Universal/GlobalE
Universal/GlobalE
