# Supplementary material for: Culicoides Midge Abundance across Years: Modeling Inter-Annual Variation for an Avian Feeder and a Candidate Vector of Hemorrhagic Diseases in Farmed Wildlife
Source: Viruses. 2024 May 11;16(5):766. doi: 10.3390/v16050766 (PMC11125994; doi:10.3390/v16050766)
Supplement: Supplementary file 1 [file viruses-16-00766-s001.zip › viruses-2876802-Supplementary.pdf]

Supplementary Materials

Table S1. Pearson correlation r values for assessing correlation between continuous covariates used to model *Culicoides* spp. weekly abundance on a wildlife ranch in the Florida panhandle.

		Latitude	Longitude	Feeder	Water	UD
2015	Latitude	1.0000	0.2570	0.5661	-0.0282	0.1504
	Longitude	0.2570	1.0000	0.2170	-0.5426	-0.3158
	Feeder	0.5661	0.2170	1.0000	0.3105	-0.1633
	Water	-0.0282	-0.5426	0.3105	1.0000	-0.0880
	UD	0.1504	-0.3158	-0.1633	-0.0880	1.0000
2016	Latitude	1.0000	0.2570	0.5661	-0.0282	0.1504
	Longitude	0.2570	1.0000	0.2170	-0.5426	-0.2256
	Feeder	0.5661	0.2170	1.0000	0.3105	-0.0451
	Water	-0.0282	-0.5426	0.3105	1.0000	-0.0331
	UD	-0.0789	-0.2256	-0.0451	-0.0331	1.0000

Table S2. ANOVA *p*-values for testing correlation between continuous numerical and categorical variables used to model *Culicoides* spp. weekly abundance on a wildlife ranch in the Florida panhandle.

	p	
**Variables**	**2015**	**2016**
Feeder vs. Habitat	6.51E-15	7.46E-23
Water vs. Habitat	3.82E-29	8.97E-45
UD vs. Habitat	6.67E-09	2.36E-04
Feeder vs. Soil	3.05E-09	2.11E-13
Water vs. Soil	2.32E-53	7.76E-81
UD vs. Soil	0.4408	0.9796

Table S3. List of models that were run to compare how selected variables relate to *Culicoides* midge preferences by species, physiological state, and year.

	Variable							
**Model ID**	**Week**	**Lat**	**Long**	**Hab**	**Feeder**	**Water**	**Soil**	**UD**
GlobalA	X	X	X	X	X	X	-	X
B1	X	X	X	X	-	X	-	X
B2	X	X	X	X	X	-	-	X
B3	X	X	X	X	X	X	-	-
B4	X	X	X	-	X	X	-	X
C1	X	X	X	X	-	-	-	X
C2	X	X	X	-	-	X	-	X
C3	X	X	X	X	-	X	-	-
C4	X	X	X	-	X	-	-	X
C5	X	X	X	X	X	-	-	-
C6	X	X	X	-	X	X	-	-
D1	X	X	X	-	X	-	-	-
D2	X	X	X	-	-	X	-	-

D3	X	X	X	X	-	-	-	-
D4	X	X	X	-	-	-	-	X
GlobalE/Universal	X	X	X	X	X	X	X	X
F1	X	X	X	X	-	X	X	X
F2	X	X	X	X	X	-	X	X
F3	X	X	X	X	X	X	X	-
F4	X	X	X	-	X	X	X	X
G1	X	X	X	X	-	-	X	X
G2	X	X	X	-	-	X	X	X
G3	X	X	X	X	-	X	X	-
G4	X	X	X	-	X	-	X	X
G5	X	X	X	X	X	-	X	-
G6	X	X	X	-	X	X	X	-
H1	X	X	X	-	X	-	X	-
H2	X	X	X	-	-	X	X	-
H3	X	X	X	-	-	-	X	X
H4	X	X	X	X	-	-	X	-
I1	X	X	X	-	-	-	X	-

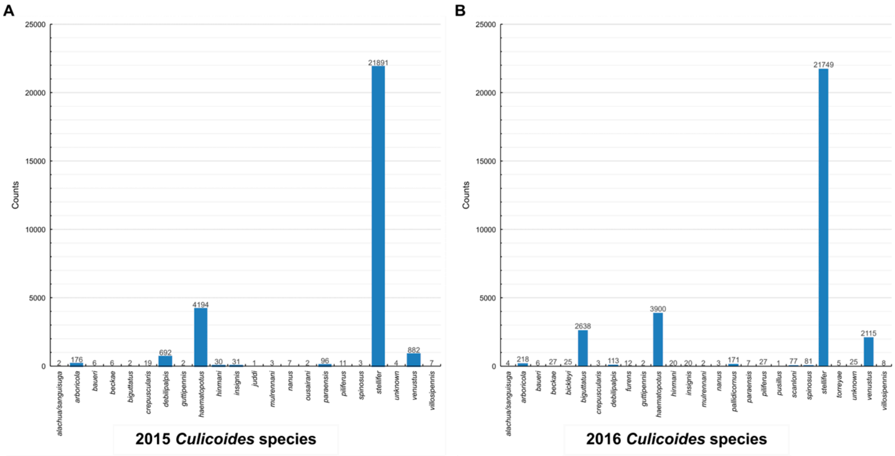

Figure S1. The total number of *Culicoides* midges collected and identified by species during the 2015 (A) and 2016 (B) sampling seasons on a wildlife ranch in the Florida panhandle.

Table S4. Summary of midge samples identified to species and physiological state that were collected from a wildlife ranch in the Florida panhandle between July – October 2015 and May – October 2016.

	Physiological state	*C. haematopotus*	*C. stellifer*	*C. venustus*	Totals
**2015 July - Oct**	Total For Species	4,548	23,266	1,073	28,887
	Total Number of Females	4,194	21,891	882	26,967
	Parous	2,421	10,471	24	12,916
	Gravid	980	5,036	331	6,347
	Bloodfed	35	1,117	17	1,169
	Nulliparous	758	5,267	510	6,535
**2016 May - Oct**	Total For Species	4,192	22,820	2,284	29,296
	Total Number of Females	3,900	21,749	2,115	27,764
	Parous	2,403	7,592	92	10,087
	Gravid	948	6,216	682	7,846
	Bloodfed	14	522	20	556
	Nulliparous	535	510	1,321	2,366

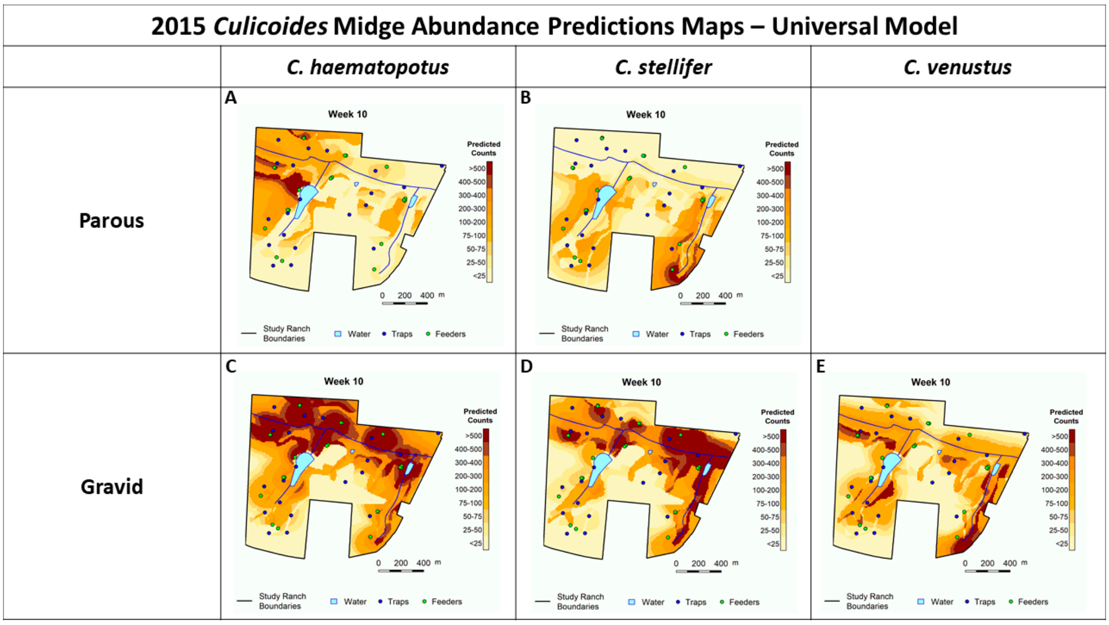

Figure S2. Still image of GIFs included in the supplemental PowerPoint file illustrating the predicted parous and gravid *Culicoides* abundance on a wildlife ranch in the Florida panhandle during the 2015 hemorrhagic disease transmission season using the universal model to make predictions for parous *C. haematopotus* (A) and *C. stellifer* (B), and gravid *C. haematopotus* (C), *C. stellifer* (D), and *C. venustus* (E).

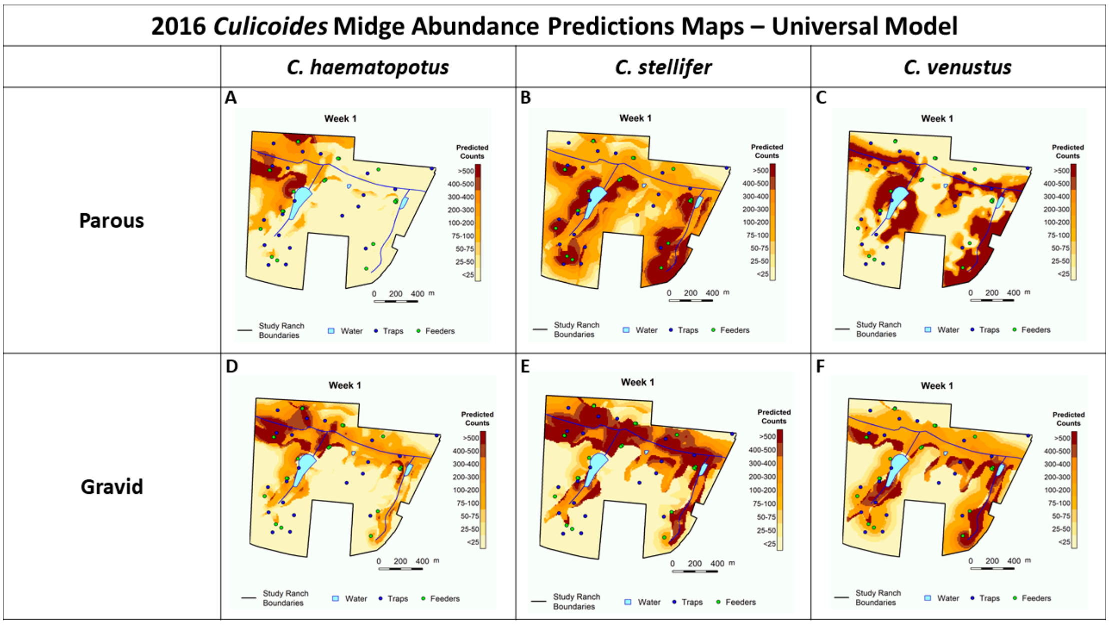

Figure S3. Still image of GIFs included in the supplemental PowerPoint file illustrating the predicted parous and gravid *Culicoides* abundance on a wildlife ranch in the Florida panhandle during the 2016 hemorrhagic disease transmission season using the universal model to make predictions for parous *C. haematopotus* (A), *C. stellifer* (B), and *C. venustus* (C), and gravid *C. haematopotus* (D), *C. stellifer* (E), and *C. venustus* (F).

3.4 Supplementary Results

3.4.1. Individual Best Models for Culicoides Species Abundance

We applied the 31 alternative models to evaluate three *Culicoides* species at the parous and gravid physiological states for two years and encountered differences in the resulting best models for each of the 12 situations. The universal model identified in the main text allowed us to draw direct comparisons among species, physiological state, and year. It was considered the best model for 2015 *C. haematopotus* gravid, 2016 *C. haematopotus* parous and gravid, and 2016 *C. stellifer* gravid but it factored in all covariates when predicting *Culicoides* midge abundance for different species, physiological states and years. In Table 4 we state the individual best models (ΔAIC=0) for those situations in which the universal model was not the most parsimonious and report the covariate estimates in Table S3.

The patterns observed in the individual best models are like those observed in the universal model with minor differences in the estimated coefficients for each year, species and physiological state for which the universal model was not the most parsimonious. The greatest deviations between the universal model and the individual best models can be best explained by which covariates were excluded from the individual best models.

For *C. haematopotus*, the universal model was also the best model for 2015 gravid, and 2016 parous and gravid. The best model for *C. haematopotus* parous in 2015 excluded covariates for distance to feeder and

UD, potentially suggesting that habitat type is more important for predicting abundance than availability of blood meals.

For *C. stellifer*, the universal model was also the best model for 2016 gravid, while 2015 parous and gravid, and 2016 parous had alternative best models. Both best models for *C. stellifer* parous and gravid in 2015 excluded the soil covariate. *C. stellifer* parous in 2015 also excluded the UD covariate while gravid in 2015 excluded the distance to water covariate. The individual best model for *C. stellifer* parous in 2016 excluded all habitat type covariate hinting that host availability and/or proximity to water are stronger determinants of abundance.

All habitat type covariates were also excluded from 2016 *C. venustus* parous, as were the covariates for distance to feeder and soil. As mentioned in the main text, the limited sample size for *C. venustus* parous in 2015 did not allow for representative modeling or predictions, and it is possible that the lesser number of significant covariates identified by the individual best model for *C. venustus* parous in 2016 are also a result of a limited sample size.

Lastly, the individual best models for *C. venustus* gravid in 2015 and 2016 both excluded covariates related to host availability. *C. venustus* gravid in 2015 excluded the distance to feeder covariate, while *C. venustus* gravid in 2016 excluded the UD covariate. Like the best model results for *C. haematopotus* parous in 2015, this suggests that habitat type is more important for predicting abundance than availability of blood meals.

Estimated covariates for the individual best models discussed here were also used to spatially predict the abundance of *C. haematopotus, C. stellifer* and *C. venustus* at the parous and gravid physiological states. We have displayed the spatial predictions for the 14th week of the 2015 and 2016 HD seasons in Figure S4 (parous) and Figure S5 (gravid), with GIFs for the entire season animated in Figure S6 and Figure S7. Graphs displaying the actual counts compared to the predicted counts using the individual best model are also included in Figure S8 and Figure S9.

Table S5. Individual best models of abundance for three species of *Culicoides* in the parous and gravid physiological states based on sampling during the hemorrhagic disease (HD) season in 2015 and 2016. Covariate estimates with blank cells were not included in the selected individual best model for the specific year, species, and physiological state, and estimates with shaded cells are identical to those reported in Table 5. Counts for parous *C. venustus* were too low to use for predictions in 2015.

			2015			2016		
	**Physiological state**	**Variable**	**Estimate**	**SE**	***p* - Value**	**Estimate**	**SE**	***p* - Value**
C. haematopotus	Parous	Intercept	3.7198	0.9861	0.0002	1.3792	0.4955	0.0054
		Week	-0.0237	0.0373	0.5260	-0.0565	0.0166	0.0007
		Latitude	-0.4859	0.2082	0.0196	-0.7474	0.2029	0.0002
		Longitude	1.0658	0.2344	5.45E-06	1.0429	0.2338	8.16E-06
		Hardwood Pine	-1.1973	0.7814	0.1250	0.0905	0.7408	0.9030
		Mixed Bottomland Hardwoods	-1.0778	0.6191	0.0817	0.5947	0.5359	0.2670
		Rural/Developed/Pasture	-3.6790	0.6994	1.44E-07	-2.6068	0.7136	0.0003
		Distance to Feeder				-0.7749	0.2743	0.0047
		Distance to Water	-0.8795	0.2809	0.0017	-0.7226	0.3715	0.0517
		Soil – Well Drained	1.5339	0.4573	0.0008	1.7574	0.4300	4.37E-05
		UD				-0.8701	0.3183	0.0063
	Gravid	Intercept	4.7592	0.5340	5.01E-19	1.8442	0.4737	9.91E-05
		Week	-0.0728	0.0207	0.0004	-0.0422	0.0138	0.0022
		Latitude	0.1728	0.1656	0.2970	-0.2125	0.1659	0.2000
		Longitude	0.4277	0.1977	0.0305	0.5066	0.2375	0.0329
		Hardwood Pine	0.7949	0.5634	0.1580	0.7308	0.6564	0.2660
		Mixed Bottomland Hardwoods	0.6951	0.3638	0.0560	1.5035	0.4125	0.0003
		Rural/Developed/Pasture	-1.9882	0.4918	5.29E-05	-2.8471	0.9230	0.0020
		Distance to Feeder	-0.6294	0.1941	0.0012	-0.8668	0.2452	0.0004
		Distance to Water	-0.6552	0.2829	0.0206	-1.2174	0.3917	0.0019
		Soil – Well Drained	0.7038	0.3418	0.0395	1.0071	0.3265	0.0020
		UD	-0.2136	0.1416	0.1320	-0.3294	0.2568	0.1990
C. stellifer	Parous	Intercept	4.4694	0.4606	2.93E-22	4.2131	0.1972	3.12E-101
		Week	0.0256	0.0201	0.2020	-0.0569	0.0105	6.36E-08
		Latitude	0.2336	0.1377	0.0898	-0.0800	0.1151	0.4870
		Longitude	-0.319	0.1331	0.0165	-0.4304	0.1125	0.0001
		Hardwood Pine	-1.919	0.4314	0.0000			
		Mixed Bottomland Hardwoods	-1.2849	0.3109	0.0000			
		Rural/Developed/Pasture	-0.9044	0.3248	0.0054			
		Distance to Feeder	-0.2305	0.1262	0.0678	-0.7951	0.1234	1.18E-10
		Distance to Water	-0.5077	0.1176	1.58E-05	-0.6866	0.1288	9.86E-08
		Soil – Well Drained				1.0380	0.2645	8.70E-05
		UD				-0.1458	0.0983	0.1380
	Gravid	Intercept	4.4514	0.4944	2.19E-19	2.5868	0.2670	3.43E-22
		Week	-0.0281	0.0227	0.2150	-0.0650	0.0093	2.77E-12
		Latitude	0.5731	0.1305	1.13E-05	0.0947	0.0965	0.3270
		Longitude	0.1022	0.1346	0.4480	0.2074	0.1102	0.0599
		Hardwood Pine	-0.3038	0.4597	0.5090	0.5949	0.4261	0.1630
		Mixed Bottomland Hardwoods	1.3891	0.3259	2.03E-05	2.5083	0.2470	3.12E-24
		Rural/Developed/Pasture	-1.9397	0.3450	1.88E-08	-0.9730	0.2990	0.0011
		Distance to Feeder	-0.7445	0.1257	3.14E-09	-0.6411	0.1191	7.37E-08
		Distance to Water				-0.6112	0.1529	6.39E-05

		Soil – Well Drained				0.8486	0.2148	7.79E-05
		UD	-0.3071	0.0996	0.0020	-0.1660	0.0899	0.0647
C. venustus	Parous	Intercept				3.4918	0.7200	0.0000
		Week				-0.3808	0.0912	2.99E-05
		Latitude				-0.0543	0.5394	0.9200
		Longitude				-1.3027	0.5759	0.0237
		Hardwood Pine						
		Mixed Bottomland Hardwoods						
		Rural/Developed/Pasture						
		Distance to Feeder						
		Distance to Water				-2.8149	0.8848	0.0015
		Soil – Well Drained						
		UD				-1.9061	0.7632	0.0125
	Gravid	Intercept	3.1775	0.7542	2.52E-05	2.3450	0.5068	3.70E-06
		Week	-0.0349	0.0213	0.1010	-0.0435	0.0133	0.0011
		Latitude	-0.2615	0.1322	0.0478	0.0435	0.1093	0.6910
		Longitude	-0.7859	0.1782	1.03E-05	-0.6766	0.1446	2.87E-06
		Hardwood Pine	1.4557	0.6011	0.0155	0.0383	0.8729	0.9650
		Mixed Bottomland Hardwoods	1.399	0.3732	0.0002	2.3582	0.3176	1.13E-13
		Rural/Developed/Pasture	-0.4192	0.4378	0.3380	-0.0885	0.3913	0.8210
		Distance to Feeder				-0.5485	0.1522	0.0003
		Distance to Water	-1.1999	0.2222	6.66E-08	-0.7962	0.1949	4.41E-05
		Soil – Well Drained	1.2005	0.3200	0.0002	1.4393	0.2743	1.55E-07
		UD	-0.4656	0.1817	0.0104			

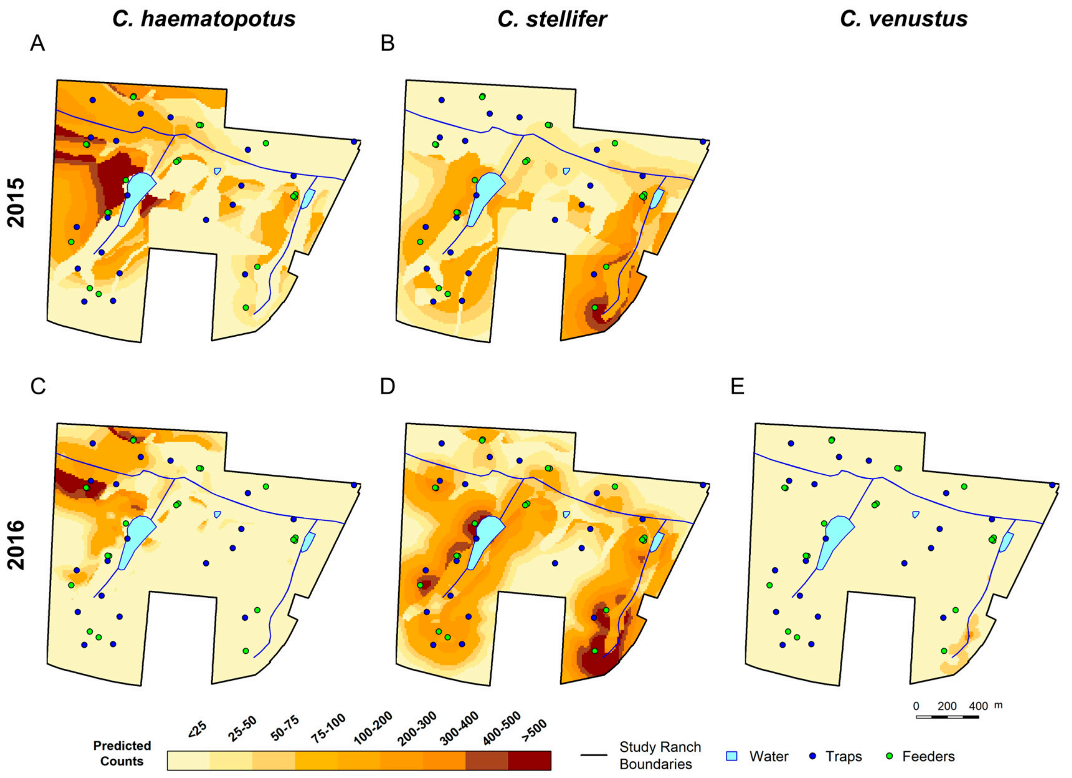

Figure S4. Predicted parous *Culicoides* abundance on a wildlife ranch in the Florida panhandle during the 14th week (8/3/2015-8/7/2015, 8/1/2016-8/5/2016) of the sampling season using the individual best model for each year, species, and physiological state to make predictions for *C. haematopotus* (A) and *C. stellifer* (B) in 2015, and predictions for *C. haematopotus* (C), *C. stellifer* (D), and *C. venustus* (E) in 2016. Counts for *C. venustus* were too low to use for spatial predictions in 2015.

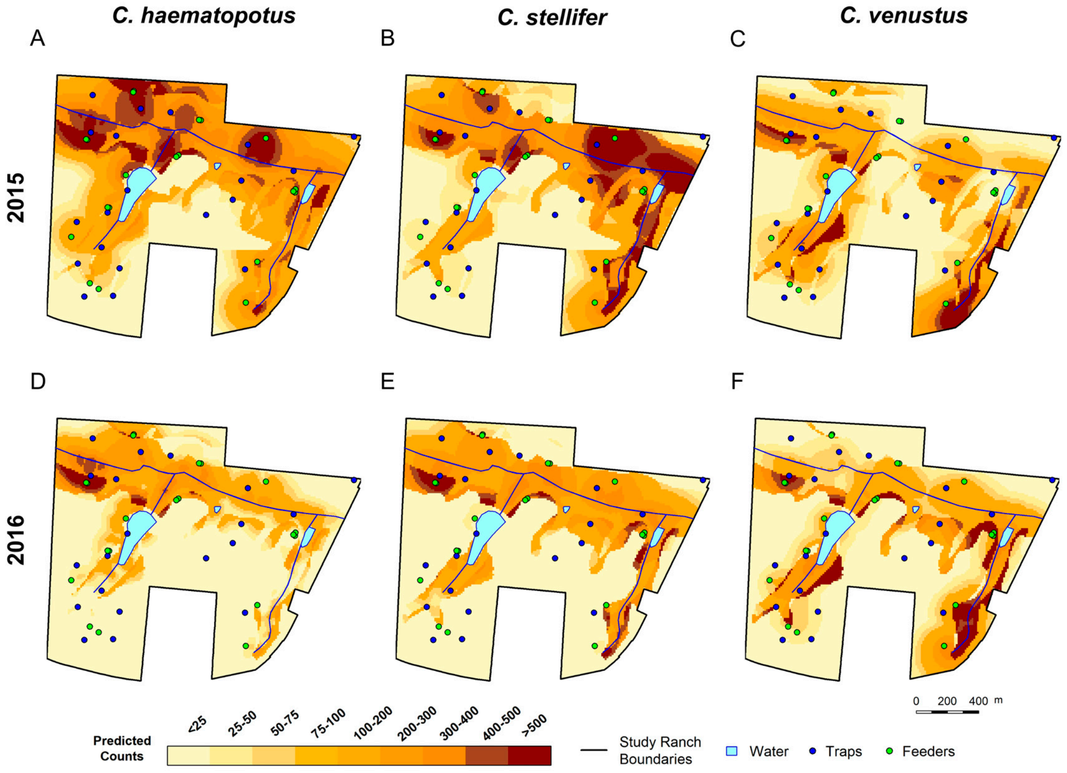

Figure S5. Predicted gravid *Culicoides* abundance on a deer ranch in the Florida panhandle during the 14th week (8/3/2015-8/7/2015, 8/1/2016-8/5/2016) of the HD transmission season using the individual best model for each year, species and physiological state to make predictions for *C. haematopotus* (A), *C. stellifer* (B), and *C. venustus* (C) in 2015, and predictions for *C. haematopotus* (D), *C. stellifer* (E), and *C. venustus* (F) in 2016.

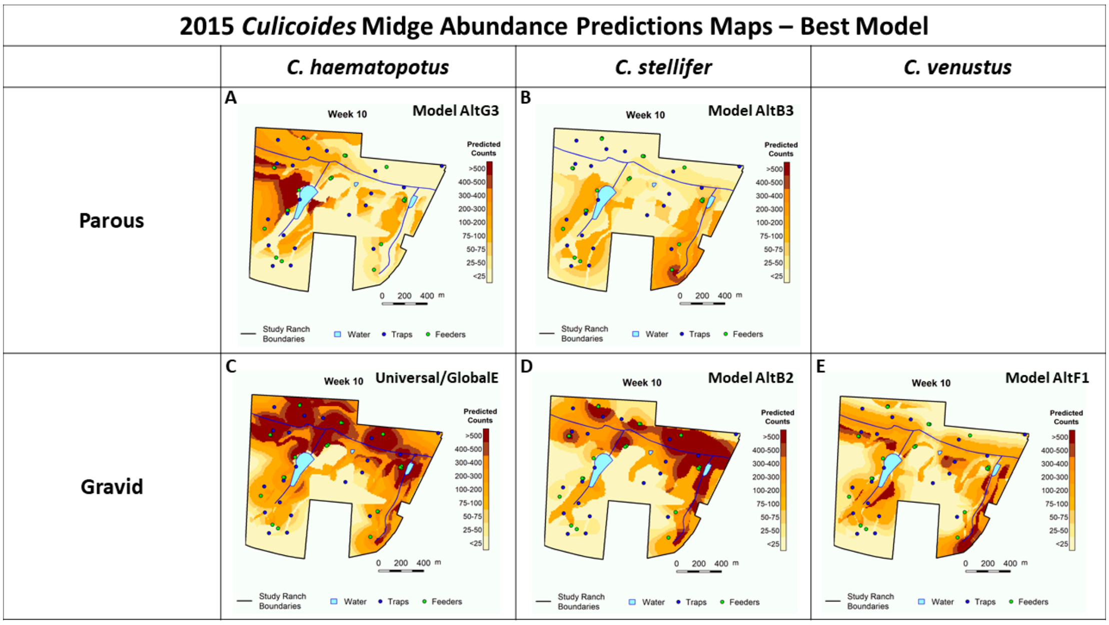

Figure S6. Still image of GIFs included in the supplemental PowerPoint file illustrating the predicted parous and gravid *Culicoides* abundance on a wildlife ranch in the Florida panhandle during the 2015 hemorrhagic disease transmission season. Maps were created using the individual best model for each year, species, and physiological state to make predictions for parous *C. haematopotus* (A) and *C. stellifer* (B), and gravid *C. haematopotus* (C), *C. stellifer* (D), and *C. venustus* (E). Best model IDs are noted in each panel and correspond to Table 4, and Table S1, with covariate estimates displayed in Table S5.

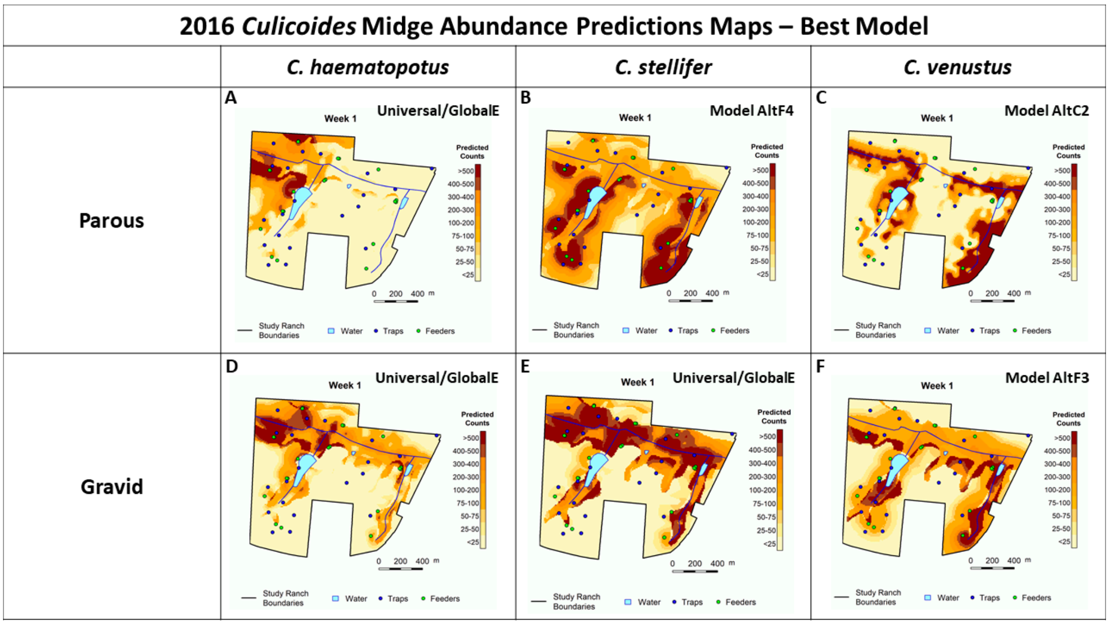

Figure S7. Still image of GIFs included in the supplemental PowerPoint file illustrating the predicted parous and gravid *Culicoides* abundance on a wildlife ranch in the Florida panhandle during the 2016 hemorrhagic disease transmission season. Maps were created using the individual best model for each year, species, and physiological state to make predictions for parous *C. haematopotus* (A), *C. stellifer* (B), and *C. venustus* (C), and gravid *C. haematopotus* (D), *C. stellifer* (E), and *C. venustus* (F). Best model IDs are noted in each panel and correspond to Table 4, and Table S1, with covariate estimates displayed in Table S5.

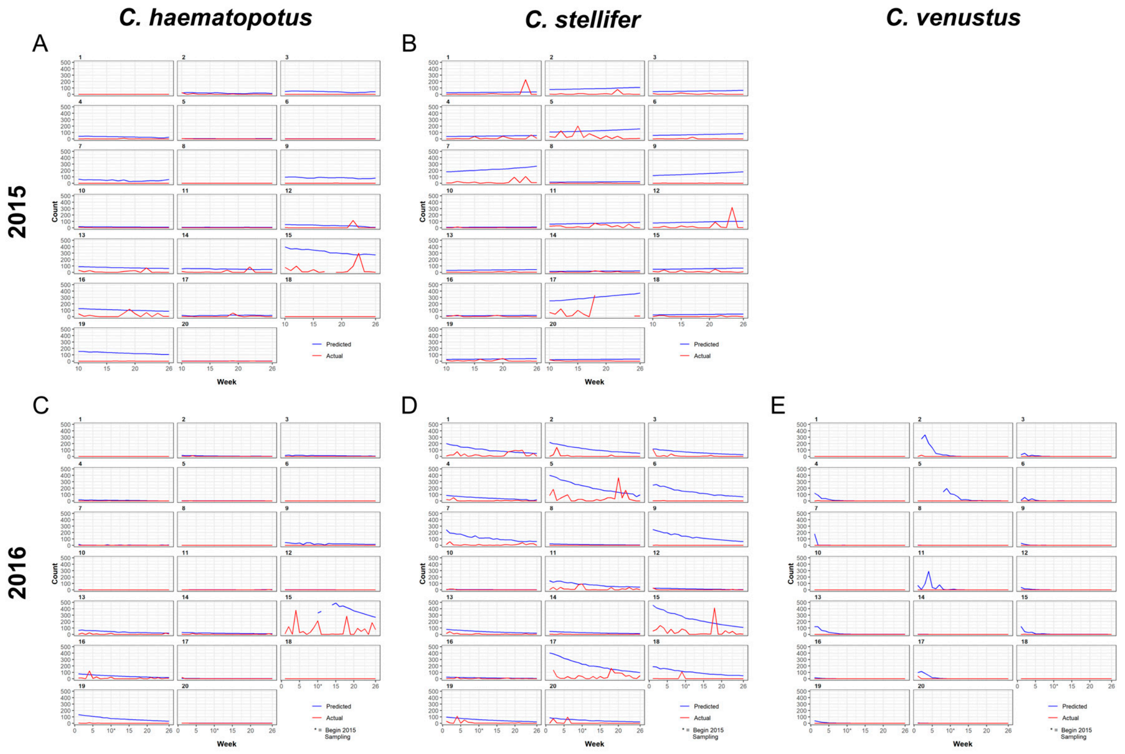

Figure S8. Predicted parous midge counts according to the individual best model compared to the actual midge counts observed during the hemorrhagic disease season on a wildlife ranch in the Florida panhandle presented by week and trap number with *C. haematopotus* (A) and *C. stellifer* (B) data from 2015, and *C. haematopotus* (C), *C. stellifer* (D), and *C. venustus* (E) data from 2016. Counts for *C. venustus* were too low to use for spatial predictions in 2015.

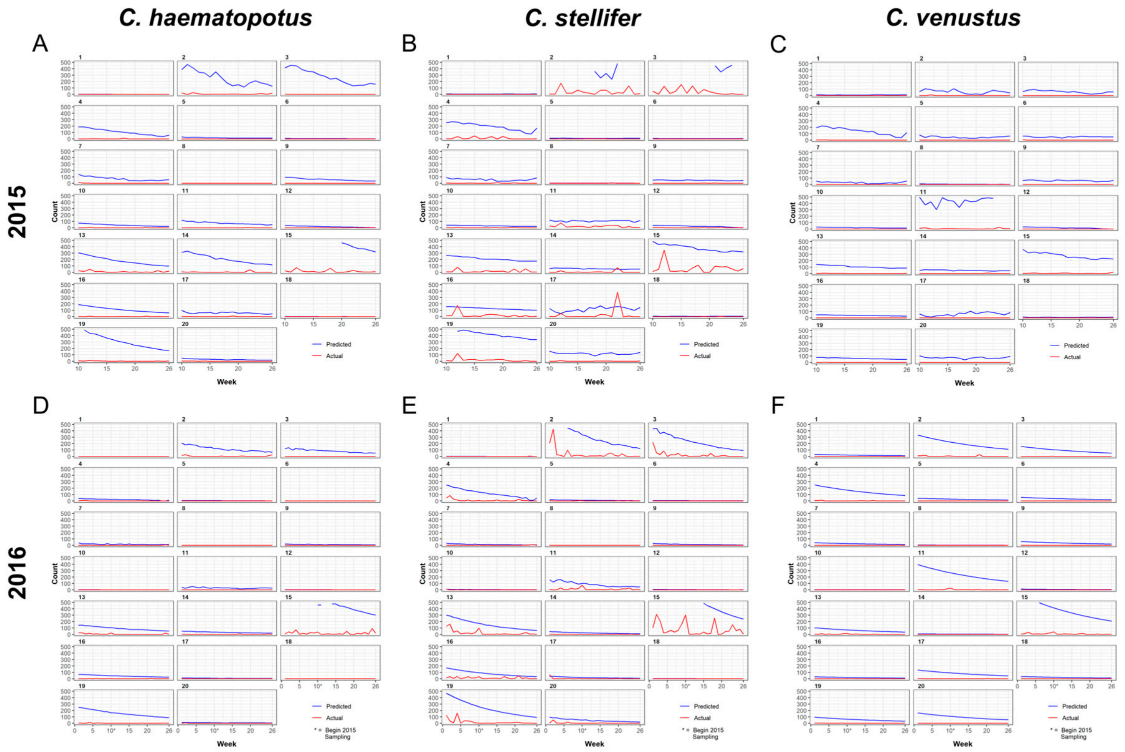

Figure S9. Predicted gravid midge counts according to the individual best model compared to the actual midge counts observed during the hemorrhagic disease season on a wildlife ranch in the Florida panhandle presented by week and trap number with *C. haematopotus* (A), *C. stellifer* (B), and *C. venustus* (C) data from 2015, and *C. haematopotus* (D), *C. stellifer* (E), and *C. venustus* (F) data from 2016.
